# MNMO: discover driver genes from a multi-omics data based-multi-layer network

**DOI:** 10.1093/bioinformatics/btaf134

**Published:** 2025-03-27

**Authors:** Zheng Deng, Jingli Wu, Xiaorong Chen, Gaoshi Li, Jiafei Liu, Zhipeng Hu, Rongyuan Li, Wansu Deng

**Affiliations:** Guangxi Key Lab of Multi-source Information Mining & Security, Guangxi Normal University, Guilin 541004, China; College of Computer Science and Information Engineering, Guangxi Normal University, Guilin 541004, China; Guangxi Key Lab of Multi-source Information Mining & Security, Guangxi Normal University, Guilin 541004, China; College of Computer Science and Information Engineering, Guangxi Normal University, Guilin 541004, China; Key Lab of Education Blockchain and Intelligent Technology, Ministry of Education, Guangxi Normal University, Guilin 541004, China; College of Computer, National University of Defense Technology, Changsha 410073, China; Guangxi Key Lab of Multi-source Information Mining & Security, Guangxi Normal University, Guilin 541004, China; College of Computer Science and Information Engineering, Guangxi Normal University, Guilin 541004, China; Key Lab of Education Blockchain and Intelligent Technology, Ministry of Education, Guangxi Normal University, Guilin 541004, China; Guangxi Key Lab of Multi-source Information Mining & Security, Guangxi Normal University, Guilin 541004, China; College of Computer Science and Information Engineering, Guangxi Normal University, Guilin 541004, China; Key Lab of Education Blockchain and Intelligent Technology, Ministry of Education, Guangxi Normal University, Guilin 541004, China; Guangxi Key Lab of Multi-source Information Mining & Security, Guangxi Normal University, Guilin 541004, China; College of Computer Science and Information Engineering, Guangxi Normal University, Guilin 541004, China; Guangxi Key Lab of Multi-source Information Mining & Security, Guangxi Normal University, Guilin 541004, China; College of Computer Science and Information Engineering, Guangxi Normal University, Guilin 541004, China; Department of Radiopharmaceuticals, School of Pharmacy, Nanjing Medical University, Nanjing 211166, China

## Abstract

**Motivation:**

Cancer as a public health problem is driven by genomic variations in “cancer driver” genes. The identification of driver genes is critical for the discovery of key biomarkers and the development of personalized therapy.

**Results:**

We propose a prediction method MNMO: a multi-layer network model based on multi-omics data. MNMO firstly constructs a dynamically adjusted four-layer network composed of miRNAs and three kinds of genes with different features. Then three kinds of scores, i.e. control capacity, mutation score, and network score, are devised and calculated by harmonic mean to produce the integrated gene score. Experiments were performed on three kinds of real cancer data to compare the identification performance of method MNMO with that of six state-of-the-art ones. The results indicate that method MNMO presents the best identification performance under most circumstances. The genes prioritized by method MNMO not only have a better match to the benchmark ones than those identified by the other methods, but also are all associated with the development and progression of cancers. In addition, some extended versions of method MNMO can further achieve better performance on most evaluation metrics for some specific datasets. They may be more conducive to identifying tissue-specific genes, which has been verified through a number of experiments.

**Availability and implementation:**

The source code and the R package “MNMO” are available at https://github.com/Zheng-D/MNMO. The dataset and code are archived at https://doi.org/10.5281/zenodo.14969986.

## 1 Introduction

Driver mutations that confer selective survival advantages on tumor cells have been acknowledged as the cause of cancer. With the development of sequencing technology, a large number of cancer genomic data have been accumulated by some large-scale cancer projects, such as The Cancer Genome Atlas (TCGA) (Tomczak *et al.* 2015) and the International Cancer Genome Association (ICGC) (Hudson *et al.* 2010). Research can be performed to identify driver genes that contain driver mutations by taking advantage of the massive data.

Early methods for identifying driver genes are mainly based on the frequency of gene mutation. They compared the mutation rates of individual genes with the background mutation rate, so as to pick out the genes mutating in a great number of cancer patients rather than by chance (Lawrence *et al.* 2013). With the development of sequencing technology, more other omics data are adopted to improve the identification performance. The interaction between genes has thrown a new light upon the mechanism of gene mutations. The genetic aberration within a biological network may lead to network architectural changes (Cheng *et al.* 2016), which may drive cells to new phenotypic states and lead to the development of cancers (Cheng *et al.* 2014). Based on those discoveries, a group of network-based methods have been put forward. Some ones (Hofree *et al.* 2013, Jia and Zhao 2014) map the mutated genes to a gene–gene interaction network, and identify driver genes by extracting significantly mutated subnetworks. Some ones (Bashashati *et al.* 2012, Hou and Ma 2014) conduct identification from a bipartite graph assuming that candidate driver genes tend to affect a large number of differentially expressed genes.

Due to the limits of high-throughput experiments, noise are inevitable in biological networks, i.e. significant amounts of false positive interactions exist. Therefore, different kinds of omics data have been tried to be integrated with it to alleviate the over-reliance on the network. EntroRank (Song *et al.* 2019a) identified driver genes by integrating the subcellular localization and mutual exclusive of variation frequency into the network. In 2022, a graph convolutional network-based semi-supervised learning method (Azadifar and Ahmadi 2022) was proposed using three feature vectors of each gene. In 2023, a graph convolution network model on a gene-miRNA network (Peng *et al.* 2023) was presented to learn the gene feature representations by aggregating the features of their neighboring miRNA nodes. In 2024, IMI-Driver (Shi *et al.* 2024) integrated multi-level network embedding and machine learning to predict driver genes. HWC (Li *et al.* 2024) applied a hierarchical weak consensus model to fusing multiple features according to the connection among them.

In addition, heat diffusion technique has also been widely used in network-based methods, trying to restore some missing interactions, or smooth the mutation frequencies across the network. NetICS (Dimitrakopoulos *et al.* 2018) conducted a per-sample bidirectional network diffusion process to capture the directionality of interactions. Subdyquency (Song *et al.* 2019b) applied a random walk process to integrate the information of subcellular localization, variation frequency and its interaction with other dysregulated genes. mND (Di Nanni *et al.* 2020) quantified gene relevance on the basis of multiple layers by using network diffusion. MinNetRank (Wei *et al.* 2020) conducted single-omics data analysis based on network diffusion, and used the minimum strategy to integrate gene mutation and differential expression data for cancer genes discovery. In 2023, a gene discovery algorithm based on two-stage random walk with restart was proposed by integrating gene expression data and gene interaction networks (Meng *et al.* 2023). In 2024, PCoDG adopted hypergraph random walks to detect cancer driver genes which cooperatively drived cancer progression in individual patients (Zhang *et al.* 2024).

As indicated above, bipartite graphs have been adopted under the cognition that driver genes directly regulate the expression of downstream dysregulated genes (Erten *et al.* 2021). However, the regulatory pathways are usually diverse, i.e. downstream dysregulated genes may be regulated indirectly by driver genes through some mediators. In this study, both direct and indirect regulations are taken into account. We begin with devising method MNMO (a Multi-layer Network model based on Multi-Omics data) by creating a multi-layer heterogeneous network, which is composed of four subnetworks constructed with miRNAs and three kinds of genes with different features, respectively. In this method, an approach is devised for quantifying control capacity of potential driver genes. A directed subnetwork is constructed by introducing a novel re-weighting process, and network diffusion is conducted to smooth the difference in mutation frequency between functionally similar genes. Then four extended versions of method MNMO are presented in terms of four kinds of network scores. Compared with six state-of-the-art methods, method MNMO and four extended versions can acquire relatively better identification performance, which has been verified through a number of experimental results.

## 2 Materials and methods

Suppose that $S_{\left|N_{S}|\times |P_{S}\right|}$ is a binary mutation matrix, $E_{\left|N_{E}|\times |P_{E}\right|}$ and ${\bar{E}}_{\left|N_{E}|\times |P_{\bar{E}}\right|}$ are two mRNA expression matrices, $I_{\left|N_{I}|\times |P_{I}\right|}$ and ${\bar{I}}_{\left|N_{I}|\times |P_{\bar{I}}\right|}$ are two miRNA expression ones. The rows of these matrices denote a group of candidate genes ($N_{S}$, $N_{E}$) or miRNAs ($N_{I}$), and the columns of them denote a group of cancer samples ($P_{S}$, $P_{E}$, $P_{I}$) or normal samples ($P_{\bar{E}}$, $P_{\bar{I}}$). In matrix S, $s_{ij}$=1 (i=1, 2, …, $\left|N_{S}\right|$, j=1, 2, …, $\left|P_{S}\right|$) if the ith gene mutates in the jth sample, and $s_{ij}$=0 otherwise. Let Γ($g_{i}$) record the set of samples in which gene $g_{i}$ mutates, i.e. Γ($g_{i}$)={$s_{-j}$ | $s_{ij}$=1}. Then the mutation frequency of gene $g_{i}$ is $\rho (g_{i})$=$\frac{\left|\Gamma (g_{i})\right|}{\left|P_{S}\right|}$. Matrices E, $\bar{E}$, I, and $\bar{I}$ are four real ones, where each entry of them represents expression level of a given gene or miRNA in a particular sample. Given an undirected protein–protein interaction (PPI) network $G_{P}$($V_{P}$, $A_{P}$), where each vertex $v_{pi}$  ∈  $V_{P}$ represents a protein generated from the expression of a gene $g_{i}$, and each undirected edge ($v_{pi}$, $v_{pj}$)∈  $A_{P}$ represents the interaction between the proteins corresponding to genes $g_{i}$ and $g_{j}$ (i  ≠  j). Therefore, it is assumed that gene $g_{i}$ also represents the vertex $v_{pi}$ in $G_{P}$. Given a bipartite graph $G_{B}$($V_{B}$, $A_{B}$) ($V_{B}$=$V_{B}^{G}$  ∪  $V_{B}^{I}$), where $V_{B}^{G}$ denotes a set of genes, and $V_{B}^{I}$ denotes a set of miRNAs. Each undirected edge ($v_{bi}$, $v_{bj}$)∈  $A_{B}$ indicates that there is an interaction between gene $v_{bi}$  ∈  $V_{B}^{G}$ and miRNA $v_{bj}$  ∈  $V_{B}^{I}$.

For each gene $g_{i}$  ∈  $N_{E}$ (resp. each miRNA $mr_{i}$  ∈  $N_{I}$), the differential expression score is evaluated with the Bhattacharyya distance, measuring the difference of gene (resp. miRNA) expression value between the normal samples and the cancer ones, as shown in Formula (1):


(1) $$de(x_{i})=\frac{{\left(\mu_{im_{1}}-\mu_{im_{2}}\right)}^{2}}{4(\sigma_{im_{1}}^{2}+\sigma_{im_{2}}^{2})}+\frac{1}{2}\ln(\frac{\sigma_{im_{1}}^{2}+\sigma_{im_{2}}^{2}}{2\sigma_{im_{1}}\sigma_{im_{2}}}),$$

where *de*($x_{i}$) denotes the differential expression score of gene $g_{i}$  ∈  $N_{E}$ (resp. miRNA $mr_{i}$  ∈  $N_{I}$). $\mu_{im_{1}}$ and $\sigma_{im_{1}}$ denote the Mean Value and the Standard Deviation of gene $g_{i}$ (resp. miRNA $mr_{i}$) expression values in normal samples, respectively. Similarly, $\mu_{im_{2}}$ and $\sigma_{im_{2}}$ denote those in cancer samples, respectively. The larger *de*($x_{i}$) is, the more apparent differential expression a gene $g_{i}$  ∈  $N_{E}$ (resp. miRNA $mr_{i}$  ∈  $N_{I}$) has.

**Figure 1.**
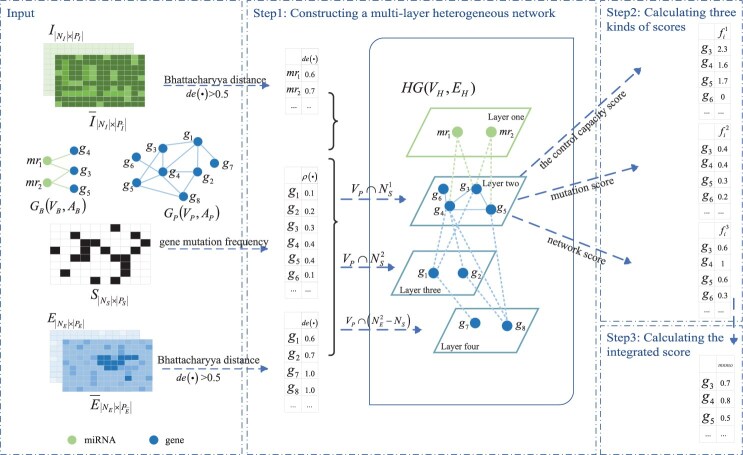
The workflow of method MNMO. Step1: Constructing a multi-layer heterogeneous network. Step2: Calculating three kinds of scores (the control capacity score, mutation score, network score). Step 3: Calculating the integrated score.

Based on the notations and definitions, a novel method MNMO is proposed. As shown in Fig. 1, a multi-layer heterogeneous network is firstly constructed. Then three kinds of scores are calculated for each potential driver gene based on the heterogeneous network. Finally, the genes are ranked in terms of the integration of the three scores. Some critical techniques of method MNMO are described as follows.

### 2.1 Constructing a multi-layer heterogeneous network

Studies have reported that mutations in driver genes would affect a large number of differentially expressed genes (Bashashati *et al.* 2012, Song *et al.* 2019b). Some of them have undergone mutations (i.e. mutated differentially expressed genes), and others have not (i.e. non-mutated differentially expressed genes). In this paper, the differentially expressed genes is determined with *de*($g_{i}$). That is to say, gene $g_{i}$  ∈  $N_{E}$ is considered as a differentially expressed gene if *de*($g_{i}$)≥  α, and is not otherwise. Let $N_{E}^{1}$ and $N_{E}^{2}$ record non-differentially expressed genes and differentially expressed ones, respectively. Then the mutation genes in set $N_{S}$ can be divided into two subsets $N_{S}^{1}$ and $N_{S}^{2}$ in terms of them, respective recording mutated non-differentially expressed genes and mutated differentially expressed ones, i.e. $N_{S}^{1}$=$N_{S}$  ∩  $N_{E}^{1}$, and $N_{S}^{2}$=$N_{S}$  ∩  $N_{E}^{2}$.

Based on the above definitions and notations, a multi-layer heterogeneous network *HG*($V_{H}$, $E_{H}$) can be built by connecting the four-layer nodes with different regulatory relationships, where each layer represents a certain type of miRNAs or genes. Nodes in layer one to layer four sequentially represent differentially expressed miRNAs, mutated non-differentially expressed genes, mutated differentially expressed genes, and non-mutated differentially expressed genes. That is to say, $V_{H}^{1}$={$mr_{i}$ | $mr_{i}$  ∈  $N_{I}$, *de*($mr_{i}$)≥  α}, $V_{H}^{2}$=$V_{P}$  ∩  $N_{S}^{1}$, $V_{H}^{3}$=$V_{P}$  ∩  $N_{S}^{2}$, $V_{H}^{4}$=$V_{P}$  ∩($N_{E}^{2}$-$N_{S}$), and $V_{H}$=$V_{H}^{1}$  ∪  $V_{H}^{2}$  ∪  $V_{H}^{3}$  ∪  $V_{H}^{4}$. Set $E_{H}$ records the edges between the nodes in different layers, which is inspired from the regulatory relationships between genes, as well as those between miRNAs and genes. It has been reported that miRNAs tend to be the regulators of cancer driver genes (Liu *et al.* 2017), and mutated cancer driver genes often retain functional miRNA binding sites, making them susceptible to miRNA inhibition (Wurm *et al.* 2023). Meanwhile, mutations in upstream driver genes have not only direct, but also indirect effects on the expression of downstream genes (Iyer *et al.* 2023). Therefore, the regulatory relationships between layer one and layer two, layer two and layer three, layer three and layer four, and layer two and layer four are constructed. Specifically, $E_{H}^{12}$={($v_{H}^{i}$, $v_{H}^{j}$)∈  $A_{B}$  |  $v_{H}^{i}$  ∈  $V_{H}^{1}$, $v_{H}^{j}$  ∈  $V_{H}^{2}$}, $E_{H}^{23}$={($v_{H}^{i}$, $v_{H}^{j}$)∈  $A_{P}$  |  $v_{H}^{i}$  ∈  $V_{H}^{2}$, $v_{H}^{j}$  ∈  $V_{H}^{3}$}, $E_{H}^{34}$={($v_{H}^{i}$, $v_{H}^{j}$)∈  $A_{P}$  |  $v_{H}^{i}$  ∈  $V_{H}^{3}$, $v_{H}^{j}$  ∈  $V_{H}^{4}$}, $E_{H}^{24}$={($v_{H}^{i}$, $v_{H}^{j}$)∈  $A_{P}$  |  $v_{H}^{i}$  ∈  $V_{H}^{2}$, $v_{H}^{j}$  ∈  $V_{H}^{4}$}. In addition, the interactions among the genes in layer-two network, i.e. $E_{H}^{22}$={($v_{H}^{i}$, $v_{H}^{j}$)∈  $A_{P}$  |  $v_{H}^{i}$, $v_{H}^{j}$  ∈  $V_{H}^{2}$}, are also recorded in set $E_{H}$. Hence, $E_{H}$=$E_{H}^{12}$  ∪  $E_{H}^{23}$  ∪  $E_{H}^{34}$  ∪  $E_{H}^{24}$  ∪  $E_{H}^{22}$. Among the genes in $V_{H}$, the ones of layer three ($V_{H}^{3}$) are regarded as intermediary genes, which may be one of the reasons that the aberrations of upstream driver genes (layer two) cause the expression changes of downstream ones (layer four) (Dimitrakopoulos *et al.* 2018). That is to say, besides the direct regulatory, the genes in $V_{H}^{2}$ also have indirect regulatory effects on the genes in $V_{H}^{4}$ through the intermediary genes in $V_{H}^{3}$. The constructed heterogeneous network is dynamically adjusted to different datasets, for a given gene may present different mutation frequencies and expression values in different datasets.

Take Fig. 1 as an example, {$mr_{1}$, $mr_{2}$}, {$g_{1}$, $g_{2}$, $g_{3}$, $g_{4}$, $g_{5}$, $g_{6}$}, and {$g_{1}$, $g_{2}$, $g_{7}$, $g_{8}$} are a group of differentially expressed miRNAs, mutated genes, and differentially expressed genes, respectively. Partition genes $g_{1}$ to $g_{8}$ into $N_{S}^{1}$, $N_{S}^{2}$, and $N_{E}^{2}$ in terms of whether they are mutated or differentially expressed, i.e. {$g_{3}$, $g_{4}$, $g_{5}$, $g_{6}$}∈  $N_{S}^{1}$, {$g_{1}$, $g_{2}$}∈  $N_{S}^{2}$, {$g_{1}$, $g_{2}$, $g_{7}$, $g_{8}$}∈  $N_{E}^{2}$. Then a multi-layer heterogeneous network *HG* is constructed. In this example, $\{mr_{1},mr_{2}\}\in V_{H}^{1}$, {$g_{3}$, $g_{4}$, $g_{5}$, $g_{6}$}∈  $V_{H}^{2}$, {$g_{1}$, $g_{2}$}∈  $V_{H}^{3}$, and {$g_{7}$, $g_{8}$}∈  $V_{H}^{4}$. The microRNA–gene and gene–gene interactions in graphs $G_{B}$ and $G_{P}$ are projected as the relationships between corresponding microRNAs (resp. genes) and genes in *HG*.

### 2.2 Measuring the control capacity with differentially expression score

Network control theory (Gao *et al.* 2014), considering how to choose a subset of network nodes to control the whole network from one state to another, has become a powerful conceptual paradigm in the field of biology to understand biological systems at a system level. The role played by each node in the subset is called control capacity (Jia and Barabási 2013), which can be quantified in many diverse ways. In this paper, the control capacity of a potential driver gene (layer two) is quantified from such aspects as the differential expressions of the downstream genes that it regulates directly and indirectly, and those of the miRNAs it interacts with. Genes with stronger control capacity means they are more likely to be driver ones.

Given a potential driver gene $g_{i}$  ∈  $v_{H}^{2}$, the differential expressions of the downstream genes that it regulates directly and indirectly are defined as in Formula (2) and (3).


(2) $$C1(g_{i})=\sum_{e_{ij}\in E_{H}^{24}}de(g_{j}),$$

(3) $$C2(g_{i})=\frac{1}{\left|e_{ij}\in E_{H}^{23}\right|}\sum_{e_{ij}\in E_{H}^{23}}\sum_{e_{jk}\in E_{H}^{34}}de(g_{k}).$$

Similarly, Formula (4) formulates the differential expressions of miRNAs with which gene $g_{i}$  ∈  $v_{H}^{2}$ interacts:


(4) $$C3(g_{i})=\sum_{e_{ji}\in E_{H}^{12}}de(mr_{j}).$$

Based on the above definition, the control capacity of gene $g_{i}$  ∈  $v_{H}^{2}$ can be calculated with Formula (5). The greater *CC*($g_{i}$) is, the stronger regulatory ability gene $g_{i}$ has.


(5) $$CC(g_{i})=\max\{C1(g_{i}),C2(g_{i})\}+C3(g_{i}).$$

Let us take gene $g_{4}$ of Fig. 1 for an example. It interacts with miRNA $mr_{1}$ in layer one, genes $g_{1}$ and $g_{2}$ in layer three, and gene $g_{8}$ in layer four. Therefore, *C1*($g_{4}$)=*de*($g_{8}$)=1, *C2*($g_{4}$)=$\frac{1}{2}$  ×(*de*($g_{7}$)+*de*($g_{8}$))=1, *C3*($g_{4}$)=*de*($mr_{1}$)=0.6, and *CC*($g_{4}$)=*max*{*C1*($g_{4}$), *C2*($g_{4}$)}+*C3*($g_{4}$)=1.6.

### 2.3 Calculating the mutation score

Given the heterogeneous network *HG*($V_{H}$, $E_{H}$), the mutation score is calculated for each gene in layer-two subnetwork. We start with weighting each edge in set $E_{H}^{22}$, then the mutation scores of genes are acquired with a network diffusion process based on random walk.

#### 2.3.1 Weighting the edges in layer-two network

For the convenience of description, let $HG^{2}$(*V2*, *E2*, *U2*, *W2*) represent the layer-two subnetwork, where $V2=V_{H}^{2}$, $E2=E_{H}^{22}$, vertex weight set *U2* records the mutation frequencies of genes, i.e. $u2_{i}=\rho (g_{i})$ and edge weight set *W2* records the similarities between genes. It is well accepted that co-expressed genes are more likely to share common biological function (Wu *et al.* 2022), hence for a pair of genes $g_{i},g_{j}\in V_{H}^{2}$, their similarity $w2_{ij}$ is ascertained with the similarity of gene expression profiles, measured with Pearson Correlation Coefficient, as follows:


(6) $$w2_{ij}=\{\begin{array}{cc} \left|\mathrm{pcc}(e_{g_{i}-},e_{g_{j}-})\right| & \text{if}\;p(e_{g_{i}-},e_{g_{j}-})<0.05,\\ 0, & \text{otherwise}, \end{array}$$

where, $e_{g_{i}-}$ and $e_{g_{j}-}$ represent the expression value of the ith and the jth gene in matrix $E_{\left|N_{E}|\times |P_{E}\right|}$, respectively. p($e_{g_{i}-}$, $e_{g_{j}-}$) is the *P*-value corresponding to the t-test statistics of $e_{g_{i}-}$ and $e_{g_{j}-}$.

**Figure 2.**
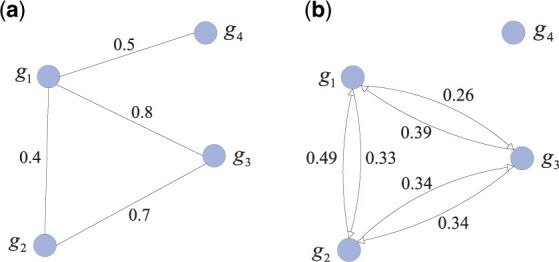
An example of the re-weighting process. (a) Initial weighted subnetwork. (b) Re-weighted subnetwork.

After the initial weighting, a re-weighting process is implemented to further quantify functional similarities between genes in layer-two subnetwork. The intuition is that a pair of genes may have higher second-order similarity if they possess more common neighbors, and are associated with their common neighbors at a similar strength simultaneously. Given a pair of genes $g_{i}$, $g_{j}$  ∈  $V_{H}^{2}$, their functional similarity $\hat{w}2_{ij}$ is computed as follows:


(7) $$\begin{array}{c} \hat{w}2_{ij}=\frac{\left|N_{g_{i}}\cap N_{g_{j}}\right|}{\left|N_{g_{i}}\right|}\times \frac{\sum_{g_{k}\in N_{g_{ij}}}\left(1-{\;\text{log}\;}_{2}\frac{w2_{ik}^{2}+w2_{jk}^{2}}{2\times w2_{ik}\times w2_{jk}}\right)}{\left|N_{g_{i}}\cap N_{g_{j}}\right|}\\ =\frac{1}{\left|N_{g_{i}}\right|}\times \sum_{g_{k}\in N_{g_{ij}}}\left(1-{\;\text{log}\;}_{2}\frac{w2_{ik}^{2}+w2_{jk}^{2}}{2\times w2_{ik}\times w2_{jk}}\right) \end{array}$$

where $N_{g_{i}}$ denotes the first-order neighbors of gene $g_{i}$, i.e. $N_{g_{i}}$=$\cup_{\forall e2_{ij}\in E2}$  $g_{j}$, similarly $N_{g_{j}}$ denotes the first-order neighbors of $g_{j}$. $N_{g_{ij}}$ records the common neighbors of $g_{i}$ and $g_{j}$. To ensure $\hat{w}2_{ij}$ falls into the range of (0,1), the edges with weight <(2–$\sqrt{3}$) are dropped from $HG^{2}$ before conducting the re-weighting process. After the re-weighting process, the edges between a pair of genes that have no common neighbors are dropped, and a new directed subnetwork $\hat{HG^{2}}$($\hat{V}2$, $\hat{E}2$, $\hat{U}2$, $\hat{W}2$) is constructed, here $\hat{V}2=V2$. Take Fig. 2 for an example, in the initial weighted subnetwork (Fig. 2a), genes $g_{1}$ and $g_{2}$ has a common neighbors $g_{3}$, then $\hat{w}2_{12}$=$\frac{1}{3}$  ×(1–${\;\text{log}\;}_{2}$  $\frac{0.8^{2}+0.7^{2}}{2\times 0.8\times 0.7}$)=0.33, and $\hat{w}2_{21}$=$\frac{1}{2}$  ×(1–${\;\text{log}\;}_{2}$  $\frac{0.8^{2}+0.7^{2}}{2\times 0.8\times 0.7}$)=0.49.

#### 2.3.2 Adjusting mutation scores through network diffusion

Given the directed subnetwork $\hat{HG^{2}}$($\hat{V}2$, $\hat{E}2$, $\hat{U}2$, $\hat{W}2$), the weights of vertices, which are initialized to the mutation frequencies of genes, are adjusted with a random walk-based network diffusion process, as equation $F_{q+1}$=β T $F_{q}$+(1– β)$F_{0}$ (Ahmed *et al.* 2020). Here $F_{0}$=($\hat{u}2_{1}$, $\hat{u}2_{2}$, …, $\hat{u}2_{\left|\hat{V}2\right|}$)${}^{T}$, recording the initial mutation frequencies of the genes in subnetwork $\hat{HG^{2}}$, is a column vector with $\left|\hat{V}2\right|$ entries. β denotes the restart probability. T represents a $\left|\hat{V}2\right|$  ×  $\left|\hat{V}2\right|$ transition matrix, each entry $t_{ij}$ denotes the probability that a simple random walk transits from ${\hat{v}}_{i}$ to ${\hat{v}}_{j}$ (${\hat{v}}_{i}$, ${\hat{v}}_{j}$  ∈  $\hat{V}2$), and is defined as the symmetric normalized form of weight ${\hat{w}}_{ij}$:


(8) $$t_{ij}=\frac{\hat{w}2_{ij}}{\sqrt{d_{i}}\cdot \sqrt{d_{j}}},$$

where $d_{i}$ as well as $d_{j}$ denote the degrees of vertices ${\hat{v}}_{i}$ and ${\hat{v}}_{j}$, respectively. This propagation function is computed iteratively until convergence, i.e. max($\left|F_{q+1}-F_{q}\right|$)<ξ. Assume that $F^{*}$ is the converged vector, then let $\hat{U}2$=$F^{*}$.

### 2.4 Calculating the integrated score

Based on the above calculation, for each gene $g_{i}$  ∈  $V_{H}^{2}$ in the layer-two network, the integrated score *mnmo*($g_{i}$) is determined as Formula (9):


(9) $$\mathrm{mnmo}(g_{i})=\{\begin{array}{cc} \frac{3}{\sum_{s=1}^{3}\frac{1}{f_{i}^{s}}}, & \text{if}\;f_{i}^{1}\neq 0,f_{i}^{2}\neq 0,\;\;\mathrm{and}\;\;f_{i}^{3}\neq 0,\\ 0, & \text{otherwise}, \end{array}$$

where $f_{i}^{1}$=*CC*($g_{i}$) denotes control capacity score, $f_{i}^{2}$=$\hat{u}2_{i}$ denotes mutation score, and $f_{i}^{3}$ denotes network score of gene $g_{i}$. It is regarded that a vertex with a high degree usually plays a significant role in biological networks, and driver genes tend to be high-degree nodes (Guo *et al.* 2018). Therefore, network score of gene $g_{i}$ is measured by the degree of gene $g_{i}$ inside the initial layer-two network. The calculation of harmonic mean on $f_{i}^{1}$, $f_{i}^{2}$, and $f_{i}^{3}$ means that it is anticipated to maximizing *mnmo*($g_{i}$) on the premise of minimizing the dispersion of each sub-score. In addition, due to the different ranges of the three sub-scores, they are rescaled into the range [0, 1] by applying the Min-Max normalization method.

### 2.5 The time complexity of method MNMO

In this section, the time complexity of method MNMO is analyzed and discussed. To simplify the description, the number of genes/miRNAs is uniformly denoted as n, and the number of samples is uniformly denoted as m. As described above, in the process of constructing the heterogeneous network, the differential expression scores of genes and miRNAs are calculated with time O (*nm*). The nodes of each layer are ascertained with time *O*($n^{2}$). During calculating the three kinds of scores, the measurement of control capacity scores takes time O($n^{3}$). The calculation of the mutation score takes time O($n^{4}$  m). Calculating the network score takes time O($n^{2}$). Finally, the integrated score is calculated with time O(n). Therefore, the total time complexity of method MNMO is O($n^{4}m$).

## 3 Comparison results and analysis

In this section, real biological datasets were applied to compare the performance of method MNMO with methods NetICS, Subdyquency, mND, EntroRank, MinNetRank, and HWC. Methods NetICS were executed on MATLAB 2018a, and methods Subdyquency, mND, MinNetRank, HWC and MNMO were executed on R 4.0.3. All of the experiments were conducted on a Lenovo workstation with Intel Core i9-12900 3.20 GHz CPU and 64GB RAM. The operating system was Windows 11.

### 3.1 Datasets

As displayed in Table 1, three experimental datasets, called Data1, Data2, and Data3, were constructed in terms of three types of cancers, i.e. breast invasive carcinoma (BRCA), prostate adenocarcinoma (PRAD), and lung adenocarcinoma (LUAD). The somatic mutation data, gene and miRNA expression data were acquired from the TCGA database. In the experiments, the raw transcriptome data were adopted for capturing more original transcriptome patterns (Han and Men 2018). The genes with missing or low (the average expression across all samples is <1) expression values were dropped from the gene expression data. The PPI network data, including 19 699 proteins and 1 858 944 interactions with “combined score” more than 400, were acquired from the STRING database (Szklarczyk *et al.* 2023). The microRNA–gene interactions with Integrated Score >0.4 were downloaded from microRNA Data Integration Portal (mirDIP) (Tokar *et al.* 2018).

**Table 1.** The number of genes and samples in the three datasets (N and C represent the normal and the cancer sample, respectively).

	Somatic mutation		Gene expression			miRNA expression		
	Gene	C	Gene	C	N	mirna	C	N
Data1	18 846	986	18 067	1091	113	2221	1103	104
Data2	12 415	495	17 770	495	52	2080	499	46
Data3	18 497	561	19 429	513	59	2197	521	46

### 3.2 Performance evaluation and parameter settings

In this experiment, the identification accuracy, measuring how well the identified driver genes match the benchmark genes, was evaluated by three widely used statistical tests, as defined in Formulas (10) to (12):


(10) $$\mathrm{Precision}=\frac{TP}{TP+FP},$$

(11) $$\mathrm{Recall}=\frac{TP}{TP+FN},$$

(12) $$F1\;\mathrm{score}=2\times \frac{\mathrm{Precision}\times \mathrm{Recall}}{\mathrm{Precision}+\mathrm{Recall}}.$$

For a given dataset, the integrated score is calculated for each gene, and the top K ranked genes were chosen as the identified driver genes. The 723 genes, obtained from Catalogue Of Somatic Mutations In Cancer (COSMIC) (Tate *et al.* 2019), were used as the benchmark driver genes.

The partial area under the *ROC* curve (*pAUC*) was used to evaluate the effect of prioritizing genes at low false positive rates (Scott and Barton 2007). It measures the number of true positives that score higher than the *n*th highest scoring negative, measured for all values from 1 to *n*:


(13) $$\mathrm{pAU}C_{n}=\frac{1}{\mathrm{nTC}}\sum_{i=1}^{n}TC_{i},$$

where *TC* is the total number of known cancer genes and $TC_{i}$ is the number of true positives that score higher than the ith highest scoring negative.

In addition, functional enrichment analysis is conducted in terms of functional consistency (*FC*), which measures the function matching degree between the detected driver genes and the benchmark genes (Erten *et al.* 2021), as follows:


(14) $$FC=\frac{\left|F(B)\cap F(M)\right|}{\left|F(B)\cup F(M)\right|},$$

where F(B) records the functions possessed by the benchmark gene set B, and F(M) records those possessed by the identified gene set M having top K genes. In this study, three commonly used databases, such as Gene Ontology (GO) (Ashburner *et al.* 2000), Kyoto Encyclopedia of Genes and Genomes (KEGG) (Kanehisa *et al.* 2017), and Reactome (Fabregat *et al.* 2018), were adopted to acquire the gene functions, respectively.

In the experiments, the parameters of methods NetICS, Subdyquency, mND, EntroRank, MinNetRank, and HWC were set as in the literatures. In the proposed MNMO method, parameter α is adopted to select differential expression genes. The identification performance with different α settings was tested and shown in Supplementary Table S1. Since method MNMO can achieve relatively good identification performance with α=0.5 or α=1.0, 0.5 was chosen for α in the following experiments. The rest parameters of method MNMO were set as follows: the restart probability β is set to 0.4 (Ahmed *et al.* 2020), and ξ is set to $10^{-6}$ (Li *et al.* 2020).

### 3.3 Comparison of identification accuracy

**Figure 3.**
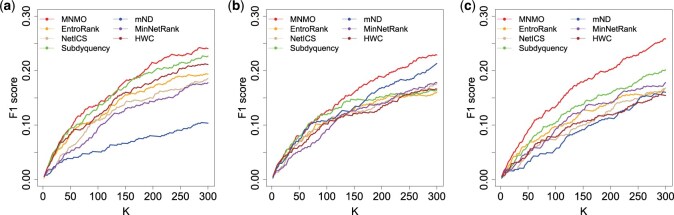
Comparison of F1 score among MNMO and other six methods. (a) BRCA. (b) PRAD. (c) LUAD.

In this section, the identification accuracy is compared among method MNMO and other six ones. Figure 3 illustrates the comparisons of F1 score on the three cancer datasets (the comparisons of *Precision* and *Recall* are shown in Supplementary Fig. S1), where K=2,4,…,300. Furthermore, for each dataset, significant tests were performed between the MNMO method and each of the comparison methods, i.e. Mann-Whitney U tests were applied to test two groups of identification accuracy under different K settings. As shown in Supplementary Tables S2–S4, the rows indicate the *P*-values between method MNMO and the corresponding comparison methods. A *P*-value of <0.05 was considered significant. From the first three columns, it is noticed that all of the *P*-values are <0.05 except for those corresponding to method Subdyquency for dataset BRCA. Therefore, for dataset BRCA, method MNMO exhibits significantly superior identification accuracy to other methods except Subdyquency, and it performs significantly better than each of the comparison approaches on datasets PRAD and LUAD.

**Figure 4.**
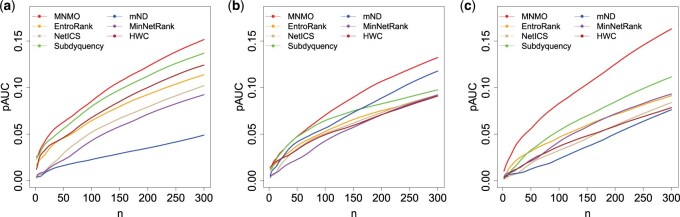
Comparison of *pAUC* among MNMO and other six methods. (a) BRCA. (b) PRAD. (c) LUAD.

In Fig. 4, the *pAUC* values are compared with different n settings, i.e. n = 2, 4, …, 300. As shown in Fig. 4a–c, the MNMO method can obtain the highest *pAUC* among the seven methods under each n setting. Similarly, Mann-Whitney U tests were conducted to test the two groups of *pAUC* values of method MNMO and a comparison method under different n settings, as shown in the last column of Supplementary Tables S2–S4. It is discovered that method MNMO presents significantly higher *pAUC* than other methods on the three datasets, for all of the *P*-values are <0.05.

In addition, Table 2 demonstrates the top 20 genes identified by the MNMO method. Most of them have been recorded in the COSMIC benchmark database except for genes *UBC*, *ACTB*, *APOB*, *ANK2*, *TLR4*, and *FN1* (marked in bold). In recent years, some reports have indicated that the six genes may be associated with the development and progression of cancers. Gene *UBC* has been suggested to be a lung cancer target (Tang *et al.* 2015). Gene *ACTB* is closely associated with a variety of cancers and de-regulated in lung and prostate (Guo *et al.* 2013). Gene *APOB* is related to the development and progression of different types of human tumors (Lin *et al.* 2024). Gene *ANK2* is a potentially favorable biomarker for LUAD prognosis (Zhang *et al.* 2022). Gene *TLR4* may play crucial roles in promoting prostate and lung cancer immune escape, survival, and metastasis (He *et al.* 2007, Pei *et al.* 2008). Gene *FN1* is significantly overexpressed in prostate cancer cell lines and human prostate cancer, and is positively associated with aggressive prostate cancer (Das *et al.* 2016).

**Table 2.** The top 20 genes identified by the MNMO method (displayed in a sorted order).^a^

Datasets	Genes									
BRCA	*TP53*	*PIK3CA*	*PTEN*	*EP300*	*CDH1*	*NCOR1*	*CREBBP*	*RUNX1*	*BRCA1*	*AKT1*
	*ATM*	*CHD4*	*KMT2C*	*PIK3R1*	*KDM6A*	*KMT2A*	*NF1*	*ATRX*	*SETD2*	***UBC***
PRAD	*TP53*	*CTNNB1*	*PTEN*	*EGFR*	*PIK3CA*	*AKT1*	*ATM*	*PTPRC*	***ACTB***	*STAT3*
	*CREBBP*	***APOB***	*KDM6A*	*SMAD4*	*CDH1*	***ANK2***	*KRAS*	***TLR4***	*MYC*	***FN1***
LUAD	*TP53*	*KRAS*	*CTNNB1*	*SMARCA4*	*CREBBP*	*ATM*	*BRCA1*	***ANK2***	*ATRX*	***ACTB***
	*PTEN*	*NF1*	*EP300*	*SETD2*	***TLR4***	*PDGFRA*	***UBC***	*EGFR*	*SMAD4*	*KMT2A*

a The genes that are not recorded in the COSMIC database are marked in bold, and those that are identified from just one of the three datasets are marked with underline.

**Table 3.** The top 20 genes identified by methods MNMO_η2, MNMO_η3, and MNMO_η4 (displayed in a sorted order).

Datasets	Methods	Genes									
BRCA	MNMO_η2	*TP53*	*PIK3CA*	*PTEN*	*CDH1*	*KMT2C*	*NCOR1*	*EP300*	*RUNX1*	*CREBBP*	*BRCA1*
		*ATM*	*CHD4*	*AKT1*	*KMT2A*	*KDM6A*	*PIK3R1*	*NF1*	*ARID1A*	*ATRX*	***PRKDC***
PRAD	MNMO_η3	*TP53*	*CTNNB1*	*PTEN*	*PIK3CA*	*ATM*	*PTPRC*	*SPOP*	*EGFR*	*KDM6A*	*KMT2A*
		*AKT1*	*CREBBP*	*STAT3*	*SMAD4*	*FOXA1*	***ACTB***	*CUL3*	*CDH1*	*KRAS*	*ATRX*
LUAD	MNMO_η4	*TP53*	*KRAS*	*SMARCA4*	*CTNNB1*	*CREBBP*	*ATM*	*ATRX*	*BRCA1*	*NF1*	*SETD2*
		***ANK2***	*PTEN*	*KMT2C*	***ACTB***	*EP300*	*KMT2A*	*PDGFRA*	*SMAD4*	***TLR4***	***UBC***

The genes that are not recorded in the COSMIC database are marked in bold, and those that are identified from just one of the three datasets are marked with underline.

### 3.4 Comparison of functional consistency

**Figure 5.**
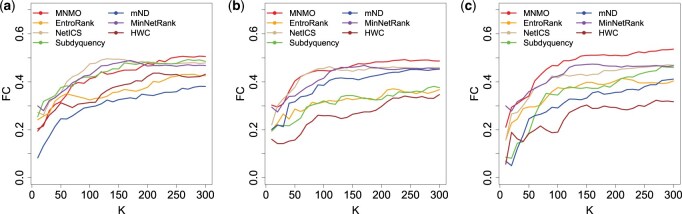
Comparison of *FC* among MNMO and other six methods based on the GO database. (a) BRCA. (b) PRAD. (c) LUAD.

In this section, functional enrichment analysis is conducted on the three cancer datasets by calculating the functional consistency (*FC*) for the top *K* candidate driver genes, where K = 2, 4, …, 300. Figure 5 illustrates the comparisons of *FC* based on the GO database. The comparison results based on the KEGG and REACTOME databases are shown in Supplementary Fig. S2. From these figures we can discover that the identified genes match better with those in the KEGG database, for the *FC* values obtained based on database KEGG are higher than those obtained based on databases GO and REACTOME. In addition, Mann-Whitney U tests were also applied to test the two groups of *FC* values of method MNMO and a comparison method under different K settings. Most of the *P*-values are <0.05, suggesting that method MNMO generally achieves significantly superior functional consistency to the other methods as far as databases GO, KEGG, and REACTOME are concerned, as shown in columns *FC*(GO), *FC*(KEGG), and *FC*(REACTOME) of Supplementary Tables S2–S4.

In addition, for each of the three datasets, enrichment analysis was conducted on the top 30 genes identified by the MNMO method, as exhibited in Supplementary Figs S3–S5. There are totally 40 GO terms, 18 KEGG pathways, and 20 REACTOME pathways are enriched by the identified genes. As illustrated in Supplementary Table S5, the enriched terms or pathways are all associated with the occurrence and progression of cancer. Take BRCA for an example, as shown in Supplementary Fig. S3, the most enriched GO term is “positive regulation of gene expression.” Regulation of gene expression affects fundamental cellular processes associated with cancer cell hallmarks (Ferlier and Coulouarn 2022). Other enriched GO terms (e.g. protein-DNA complex organization, and chromatin remodeling) are also associated with cancer. The most enriched KEGG pathway is “Human papillomavirus infection.” A growing body of evidence suggests that human papillomavirus infection may play a crucial role in invasiveness of breast cancer (Khodabandehlou *et al.* 2019). Other enriched KEGG pathways (e.g. MicroRNAs in cancer, and Thyroid hormone signaling pathway) are also related to cancer. The most enriched REACTOME pathway is “Transcriptional Regulation by TP53.” p53 is a transcription factor which suppresses tumor growth through regulating dozens of target genes with diverse biological functions (Sullivan *et al.* 2018). Other enriched REACTOME pathways (e.g. Transcriptional regulation by RUNX1, and PIP3 activates AKT signaling) are also associated with cancer.

### 3.5 Ablation experiment

In this section, a series of ablation experiments were carried out to verify the effectiveness of method MNMO using four-layer heterogeneous network and three kinds of scores.

#### 3.5.1 Analysis of multi-layer heterogeneous network

As shown in Supplementary Table S6, the identification performance with different network models was compared. Let MNMO_1 represent the method using a two-layer network, which is transformed from network *HG* by dropping the vertices in layer one ($V_{H}^{1}$) and merging those in layers two ($V_{H}^{2}$) and three ($V_{H}^{3}$). Similarly, MNMO_2 also corresponds to a two-layer network that is converted from network *HG* by dropping the vertices in layer one ($V_{H}^{1}$) and merging those in layers three ($V_{H}^{3}$) and four ($V_{H}^{4}$). MNMO_3 (resp. MNMO_4) denotes a three-layer network, which is transformed from network *HG* by merging $V_{H}^{2}$ (resp. $V_{H}^{4}$) and $V_{H}^{3}$. The comparison results demonstrate that the MNMO method performs the best under different evaluation metrics except for the *FC* based on REACTOME in the LUAD dataset.

#### 3.5.2 Analysis of three kinds of scores

In Supplementary Table S7, the detected results were illustrated under different combinations of control capacity score, mutation score, and network score. Let MNMO_C, MNMO_M, and MNMO_N represent the methods using just control capacity score, mutation score, and network score, respectively. Let MNMO_MN, MNMO_CN, and MNMO_CM denote the methods adopting two of the three scores, respectively. From this table we notice that method MNMO performs better than the other ones on datasets BRCA and LUAD, and worse than method MNMO_MN on dataset PRAD. Since method MNMO_MN denotes the one excluding control capacity score, eight datasets (five additional datasets were LIHC, LUSC, KIRP, GBM, COAD) were adopted to further analyzed the number of genes/miRNAs on each layer of the constructed multi-layer heterogeneous networks, as exhibited in Supplementary Table S8. The performance comparisons over the five additional datasets are illustrated in Supplementary Table S9. From these two tables it is discovered that datasets PRAD and COAD, on which method MNMO performs worse than method MNMO_MN, present the common feature that there are relatively fewer genes in the third and fourth layers of the heterogeneous network. The control capacity score of a gene may not be estimated effectively in the case of relatively fewer downstream genes. Therefore, the MNMO method might be more suitable for the dataset with much more genes in the third and fourth layers of the heterogeneous network.

### 3.6 Improvement of network score

Depending on the way of construction, the heterogeneous networks generated by different cancer datasets are different. For example, the number of genes in layer two is 12 518 for the BRCA dataset, 8020 for the PRAD dataset, and 11 675 for the LUAD dataset. Therefore, a certain gene of the PPI network may not always be classified into the same layer, and may have different network scores even if it belongs to the second layer in different heterogeneous networks. This may be reasonable from the perspective of tissue-specificity.

As mentioned above, the network score of gene $g_{i}$ ($g_{i}$  ∈  $v_{H}^{2}$) is calculated by its degree inside the initial layer-two network. However, due to the inevitable false positive interactions in PPI networks, the reliability of them should be considered when measuring the network scores of genes. Here the network score is further improved by taking interaction strength into account. That is to say, given the layer-two subnetwork $HG^{2}(V2,E2,U2,W2)$, $f_{i}^{3}$ is redefined as $\sum_{e2_{ij}\in E2}w2_{ij}$. In the experiments, four kinds of measurements, denoted by $\eta 1_{ij}$, $\eta 2_{ij}$, $\eta 3_{ij}$, and $\eta 4_{ij}$, were conducted for calculating $w2_{ij}$. The definition of $\eta 1_{ij}$ is the same as Formula (6), and $\eta 2_{ij}$ is evaluated with the number of common neighbors as well as the relationship among them.


(15) $$\eta 2_{ij}=\{\begin{array}{cc} \frac{1}{\left|N_{g_{ij}}\right|}\sum_{g_{k}\in N_{g_{ij}}}\frac{\left|N_{g_{k}}\cap N_{g_{ij}}\right|}{\left|N_{g_{ij}}\right|}, & \text{if }|N_{g_{ij}}|\neq 0,\\ 0, & \text{otherwise}, \end{array}$$

where $N_{g_{k}}$ denotes the first-order neighbors of gene $g_{k}$, $N_{g_{ij}}$ denotes the common neighbors of $g_{i}$ and $g_{j}$. The more common neighbors that genes $g_{i}$ and $g_{j}$ have, and the stronger connectivity among their common neighbors is, the greater $\eta 2_{ij}$ is. $\eta 3_{ij}$ (resp. $\eta 4_{ij}$) is ascertained based on the sum (resp. product) of $\eta 1_{ij}$ and $\eta 2_{ij}$.

**Figure 6.**
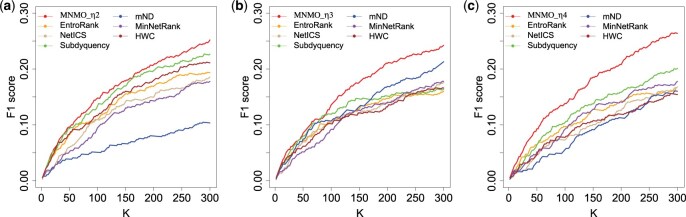
Comparison of F1 score among the extended methods and other six methods. Datasets BRCA, PRAD, and LUAD were respectively adopted for methods MNMO_η2, MNMO_η3, and MNMO_η4. (a) BRCA. (b) PRAD. (c) LUAD.

Based on the four kinds of measurements, four derived methods are produced, denoted as MNMO_η1, MNMO_η2, MNMO_η3, and MNMO_η4. The identification performance of them is tested and compared with method MNMO, as shown in Supplementary Table S10. From the table we can discover that methods MNMO_η2, MNMO_η3, and MNMO_η4 performs well for datasets BRCA, PRAD, and LUAD, respectively. Therefore, their identification accuracy was further compared with six state-of-the-art methods by using the top K candidate driver genes, where K=2, 4, …, 300. The results of F1 score are shown in Fig. 6, and those of *Precision* and *Recall* are illustrated in Supplementary Fig. S6. The proposed methods perform the best except for MNMO_η3 on the PRAD dataset with K=84 and 88.

As referred above, different heterogeneous network structures can be built based on different datasets, and different network scoring methods exhibits different performance on different datasets. In Table 3, the top 20 genes identified by methods MNMO_η2, MNMO_η3, and MNMO_η4 are listed. Compared with the genes identified by the MNMO method (as shown in Table 2), the derived methods can detect more genes which are recorded in the COSMIC benchmark database. For example, for the PRAD dataset, 19 COSMIC genes are identified by method MNMO_η3, while 15 ones are found by method MNMO. In addition, it is discovered that the derived methods can identify more dataset-specific genes, i.e. they are only detected in a specific dataset. Take the LUAD dataset for an example, method MNMO_η4 identified six specific genes, four more than method MNMO. Therefore, the improved network scores may be more conducive to identifying tissue-specific genes.

## 4 Conclusion

The identification of cancer driver genes is a hot issue in bioinformatics. In this paper, a novel identification method MNMO is proposed from integrating three omics data. It distinguishes itself from previous network-based methods by building a dynamically adjusted multi-layer heterogeneous network, and measuring the potential driver genes based on the harmonic mean of their control capacity scores, mutation scores, and network scores. In the calculation of these scores, two kinds of neighboring nodes, i.e. intra-layer neighboring nodes and inter-layer ones, are fully taken advantage of. The control capacity score of a potential driver gene is evaluated through their inter-layer neighbors, i.e. the miRNAs directly regulating it, as well as the genes directly and indirectly regulated by it. Both the mutation score and the network score of a potential driver gene are measured with the help of their intra-layer neighbors, i.e. its directly interacted genes in layer two. The worst-case running time of method MNMO is exponential in the number of genes, and the most time-consuming operation is to ascertain the mutation scores. However, since the number of genes in the second layer and the number of its internal neighbors or interacting genes/miRNAs is much smaller than the total number of genes, the method can be performed in a reasonable amount of time. Supplementary Table S11 shows the time required to run the MNMO method on the three cancer datasets.

Compared with six state-of-the-art methods, method MNMO exhibits obvious better performance in most cases. The genes prioritized by method MNMO not only match better with the benchmark genes, but also are all reported as cancer related genes in literature. During the experiments, the extended versions of method MNMO were also presented by introducing four kinds of network weighting methods. For some specific datasets, some of them outperform method MNMO in terms of most evaluation metrics, hence the extended versions may be more conducive to identifying tissue-specific genes. Nevertheless, none of the methods performs the best for each of the three datasets. In addition, it is discovered that the MNMO method might be fitter for the dataset having much more genes in layers three and four of the heterogeneous network, for its performance would be affected negatively on datasets with less genes in these two layers. Due to the reliance of scoring a gene on its neighboring nodes, the performance of method MNMO would also be limited when applying a sparse PPI network, which has less interactions among genes. It is recommended to perform some data pre-processing to alleviate the sparsity before performing method MNMO. For example, more interactions between nodes can be acquired through exploring their second-order similarity, or integrating more other omics data. Deep learning models may be adopted so as to extract network structure features from sparse dynamic biological networks, which will be studied in future work.

## Author contributions

Z.D. contributed to Conceptualization, Methodology, Software, and Writing—original draft. J.W. was involved in Conceptualization, Methodology, Funding acquisition, and Writing—review & editing. X.C., G.L., and J.L. handled Validation and Project administration. Z.H., R.L. and W.D. conducted Data curation.

## Supplementary Material

btaf134_Supplementary_Data

## Data Availability

The data and code underlying this article are available on GitHub repository ‘MNMO’, at https://github.com/Zheng-D/MNMO.

## References

[btaf134-B1] Ahmed R , Baali I, Erten C et al Mexcowalk: mutual exclusion and coverage based random walk to identify cancer modules. Bioinformatics 2020;36:872–9. 31432076 10.1093/bioinformatics/btz655

[btaf134-B2] Ashburner M , Ball CA, Blake JA et al Gene ontology: tool for the unification of biology. Nat Genet 2000;25:25–9. 10802651 10.1038/75556 PMC3037419

[btaf134-B3] Azadifar S , Ahmadi A. A novel candidate disease gene prioritization method using deep graph convolutional networks and semi-supervised learning. BMC Bioinformatics 2022;23:422. 36241966 10.1186/s12859-022-04954-x PMC9563530

[btaf134-B4] Bashashati A , Haffari G, Ding J et al Drivernet: uncovering the impact of somatic driver mutations on transcriptional networks in cancer. Genome Biol 2012;13;R124–14. 23383675 10.1186/gb-2012-13-12-r124 PMC4056374

[btaf134-B5] Cheng F , Jia P, Wang Q et al Studying tumorigenesis through network evolution and somatic mutational perturbations in the cancer interactome. Mol Biol Evol 2014;31:2156–69. 24881052 10.1093/molbev/msu167 PMC4104318

[btaf134-B6] Cheng F , Zhao J, Zhao Z. Advances in computational approaches for prioritizing driver mutations and significantly mutated genes in cancer genomes. Brief Bioinform 2016;17:642–56. 26307061 10.1093/bib/bbv068 PMC4945827

[btaf134-B7] Das DK , Naidoo M, Ilboudo A et al mir-1207-3p regulates the androgen receptor in prostate cancer via fndc1/fibronectin. Exp Cell Res 2016;348:190–200. 27693493 10.1016/j.yexcr.2016.09.021 PMC5077722

[btaf134-B8] Di Nanni N , Gnocchi M, Moscatelli M et al Gene relevance based on multiple evidences in complex networks. Bioinformatics 2020;36:865–71. 31504182 10.1093/bioinformatics/btz652 PMC9883679

[btaf134-B9] Dimitrakopoulos C , Hindupur SK, Häfliger L et al Network-based integration of multi-omics data for prioritizing cancer genes. Bioinformatics 2018;34:2441–8. 29547932 10.1093/bioinformatics/bty148 PMC6041755

[btaf134-B10] Erten C , Houdjedj A, Kazan H. Ranking cancer drivers via betweenness-based outlier detection and random walks. BMC Bioinformatics 2021;22:62–16. 33568049 10.1186/s12859-021-03989-w PMC7877041

[btaf134-B11] Fabregat A , Korninger F, Viteri G et al Reactome graph database: efficient access to complex pathway data. PLoS Comput Biol 2018;14:e1005968. 29377902 10.1371/journal.pcbi.1005968 PMC5805351

[btaf134-B12] Ferlier T , Coulouarn C. Regulation of gene expression in cancer–an overview. Cells 2022;11:4058. 36552821 10.3390/cells11244058 PMC9776464

[btaf134-B13] Gao J , Liu Y-Y, D'Souza RM et al Target control of complex networks. Nat Commun 2014;5:5415–8. 25388503 10.1038/ncomms6415 PMC4243219

[btaf134-B14] Guo C , Liu S, Wang J et al Actb in cancer. Clin Chim Acta 2013;417:39–44. 23266771 10.1016/j.cca.2012.12.012

[btaf134-B15] Guo W-F , Zhang S-W, Shi Q-Q et al A novel algorithm for finding optimal driver nodes to target control complex networks and its applications for drug targets identification. BMC Genomics 2018;19:924–79. 29363426 10.1186/s12864-017-4332-z PMC5780855

[btaf134-B16] Han H , Men K. How does normalization impact rna-seq disease diagnosis? J Biomed Inform 2018;85:80–92. 30041017 10.1016/j.jbi.2018.07.016

[btaf134-B17] He W , Liu Q, Wang L et al Tlr4 signaling promotes immune escape of human lung cancer cells by inducing immunosuppressive cytokines and apoptosis resistance. Mol Immunol 2007;44:2850–9. 17328955 10.1016/j.molimm.2007.01.022

[btaf134-B18] Hofree M , Shen JP, Carter H et al Network-based stratification of tumor mutations. Nat Methods 2013;10:1108–15. 24037242 10.1038/nmeth.2651 PMC3866081

[btaf134-B19] Hou JP , Ma J. Dawnrank: discovering personalized driver genes in cancer. Genome Med 2014;6:56–16. 25177370 10.1186/s13073-014-0056-8 PMC4148527

[btaf134-B20] Hudson TJ , Anderson W, Artez A et al, International Cancer Genome Consortium. International network of cancer genome projects. Nature 2010;464:993–8. 20393554 10.1038/nature08987 PMC2902243

[btaf134-B21] Iyer MS , Pal A, Venkatesh K. A systems biology approach to disentangle the direct and indirect effects of global transcription factors on gene expression in escherichia coli. Microbiol Spectr 2023;11:e02101–22. 36749045 10.1128/spectrum.02101-22 PMC10100776

[btaf134-B22] Jia P , Zhao Z. Varwalker: personalized mutation network analysis of putative cancer genes from next-generation sequencing data. PLoS Comput Biol 2014;10:e1003460. 24516372 10.1371/journal.pcbi.1003460 PMC3916227

[btaf134-B23] Jia T , Barabási A-L. Control capacity and a random sampling method in exploring controllability of complex networks. Sci Rep 2013;3:2354. 23912679 10.1038/srep02354 PMC3733055

[btaf134-B24] Kanehisa M , Furumichi M, Tanabe M et al Kegg: new perspectives on genomes, pathways, diseases and drugs. Nucleic Acids Res 2017;45:D353–D361. 27899662 10.1093/nar/gkw1092 PMC5210567

[btaf134-B25] Khodabandehlou N , Mostafaei S, Etemadi A et al Human papilloma virus and breast cancer: the role of inflammation and viral expressed proteins. BMC Cancer 2019;19:61. 30642295 10.1186/s12885-019-5286-0 PMC6332859

[btaf134-B26] Lawrence MS , Stojanov P, Polak P et al Mutational heterogeneity in cancer and the search for new cancer-associated genes. Nature 2013;499:214–8. 23770567 10.1038/nature12213 PMC3919509

[btaf134-B27] Li F , Gao L, Wang B. Detection of driver modules with rarely mutated genes in cancers. IEEE/ACM Trans Comput Biol Bioinform 2020;17:390–401. 29994261 10.1109/TCBB.2018.2846262

[btaf134-B28] Li G , Hu Z, Luo X et al Identification of cancer driver genes based on hierarchical weak consensus model. Health Inf Sci Syst 2024;12:21. 38464463 10.1007/s13755-024-00279-6 PMC10917728

[btaf134-B29] Lin Z , Ji X, Tian N et al Apob is a potential prognostic biomarker in hepatocellular carcinoma. Discov Oncol 2024;15:28. 38310202 10.1007/s12672-024-00877-6 PMC10838261

[btaf134-B30] Liu W , Lv C, Zhang B et al Microrna-27b functions as a new inhibitor of ovarian cancer-mediated vasculogenic mimicry through suppression of VE-cadherin expression. RNA 2017;23:1019–27. 28396577 10.1261/rna.059592.116 PMC5473136

[btaf134-B31] Meng P , Wang G, Guo H et al Identifying cancer driver genes using a two-stage random walk with restart on a gene interaction network. Comput Biol Med 2023;158:106810. 37011433 10.1016/j.compbiomed.2023.106810

[btaf134-B32] Pei Z , Lin D, Song X et al Tlr4 signaling promotes the expression of vegf and tgfβ1 in human prostate epithelial pc3 cells induced by lipopolysaccharide. Cell Immunol 2008;254:20–7. 18649875 10.1016/j.cellimm.2008.06.007

[btaf134-B33] Peng W , Wu R, Dai W et al Mirna–gene network embedding for predicting cancer driver genes. Brief Funct Genomics 2023;22:341–50. 36752023 10.1093/bfgp/elac059

[btaf134-B34] Scott MS , Barton GJ. Probabilistic prediction and ranking of human protein–protein interactions. BMC Bioinformatics 2007;8:1–21. 17615067 10.1186/1471-2105-8-239 PMC1939716

[btaf134-B35] Shi P , Han J, Zhang Y et al Imi-driver: integrating multi-level gene networks and multi-omics for cancer driver gene identification. PLoS Comput Biol 2024;20:e1012389. 39186807 10.1371/journal.pcbi.1012389 PMC11379397

[btaf134-B36] Song J , Peng W, Wang F. An entropy-based method for identifying mutual exclusive driver genes in cancer. IEEE/ACM Trans Comput Biol Bioinform 2019a;17:758–68. 30763245 10.1109/TCBB.2019.2897931

[btaf134-B37] Song J , Peng W, Wang F. A random walk-based method to identify driver genes by integrating the subcellular localization and variation frequency into bipartite graph. BMC Bioinformatics 2019b;20:238–17. 31088372 10.1186/s12859-019-2847-9 PMC6518800

[btaf134-B38] Sullivan KD , Galbraith MD, Andrysik Z et al Mechanisms of transcriptional regulation by p53. Cell Death Differ 2018;25:133–43. 29125602 10.1038/cdd.2017.174 PMC5729533

[btaf134-B39] Szklarczyk D , Kirsch R, Koutrouli M et al The string database in 2023: protein–protein association networks and functional enrichment analyses for any sequenced genome of interest. Nucleic Acids Res 2023;51:D638–46. 36370105 10.1093/nar/gkac1000 PMC9825434

[btaf134-B40] Tang Y , Geng Y, Luo J et al Downregulation of ubiquitin inhibits the proliferation and radioresistance of non-small cell lung cancer cells in vitro and in vivo. Sci Rep 2015;5:9476. 25820571 10.1038/srep09476 PMC4377628

[btaf134-B41] Tate JG , Bamford S, Jubb HC et al Cosmic: the catalogue of somatic mutations in cancer. Nucleic Acids Res 2019;47:D941–7. 30371878 10.1093/nar/gky1015 PMC6323903

[btaf134-B42] Tokar T , Pastrello C, Rossos AE et al mirdip 4.1–integrative database of human microrna target predictions. Nucleic Acids Res 2018;46:D360–70. 29194489 10.1093/nar/gkx1144 PMC5753284

[btaf134-B43] Tomczak K , Czerwińska P, Wiznerowicz M. Review the cancer genome atlas (TCGA): an immeasurable source of knowledge. Contemp Oncol Współczesna Onkologia 2015;2015:68–77. 10.5114/wo.2014.47136 PMC4322527 25691825

[btaf134-B44] Wei T , Fa B, Luo C et al An efficient and easy-to-use network-based integrative method of multi-omics data for cancer genes discovery. Front Genet 2020;11:613033. 33488678 10.3389/fgene.2020.613033 PMC7820902

[btaf134-B45] Wu J , Zhang Q, Li G. Identification of cancer-related module in protein–protein interaction network based on gene prioritization. J Bioinform Comput Biol 2022;20:2150031. 34860145 10.1142/S0219720021500311

[btaf134-B46] Wurm AA , Brilloff S, Kolovich S et al Signaling-induced systematic repression of mirnas uncovers cancer vulnerabilities and targeted therapy sensitivity. Cell Reports Med 2023;4:101200. 10.1016/j.xcrm.2023.101200 PMC10591033 37734378

[btaf134-B47] Zhang T , Zhang S-W, Xie M-Y et al Identifying cooperating cancer driver genes in individual patients through hypergraph random walk. J Biomed Inform 2024;157:104710. 39159864 10.1016/j.jbi.2024.104710

[btaf134-B48] Zhang W , Shang X, Liu N et al Ank2 as a novel predictive biomarker for immune checkpoint inhibitors and its correlation with antitumor immunity in lung adenocarcinoma. BMC Pulm Med 2022;22:483. 36539782 10.1186/s12890-022-02279-2 PMC9768990

