# Supplementary material for: MNMO: discover driver genes from a multi-omics data based-multi-layer network
Source: Bioinformatics. 2025 Mar 27;41(4):btaf134. doi: 10.1093/bioinformatics/btaf134 (PMC12033032; doi:10.1093/bioinformatics/btaf134)
Supplement: btaf134_Supplementary_Data [file btaf134_supplementary_data.zip › Supplementary Data.docx]

**SUPPLEMENTARY MATERIAL**

**MNMO: Discover driver genes from a Multi-Omics data based multi-layer network.**

Zheng Deng^2,3^, Jingli Wu^1,2,3,∗^, Xiaorong Chen^4^, Gaoshi Li^1,2,3^, Jiafei Liu^1,2,3^, Zhipeng Hu^2,3^, Rongyuan Li^2,3^ and Wansu Deng^5^

^1^Key Lab of Education Blockchain and Intelligent Technology, Ministry of Education, Guangxi Normal University, Guilin, 541004, Guangxi, China , ^2^Guangxi Key Lab of Multi-source Information Mining & Security, Guangxi Normal University, Guilin, 541004, Guangxi, China , ^3^College of Computer Science and Engineering, Guangxi Normal University, Guilin, 541004, Guangxi, China, ^4^College of Computer, National University of Defense Technology, Changsha 410073, P.R.China and ^5^Department of Radiopharmaceuticals, School of

Pharmacy, Nanjing Medical University, Nanjing 211166, P.R.China

**Supplementary Figures**

- Supplementary Figure S1. Comparison of Precision and Recall among MNMO and other six methods.

- Supplementary Figure S2. The comparison results of functional consistency on three cancer datasets.

- Supplementary Figure S3. Enrichment analysis on the top 30 genes identified by method MNMO (BRCA dataset).

-Supplementary Figure S4. Enrichment analysis on the top 30 genes identified by method MNMO (PRAD dataset).

- Supplementary Figure S5. Enrichment analysis on the top 30 genes identified by method MNMO (LUAD dataset).

- Supplementary Figure S6. Comparison of Precision and Recall among the extended methods and other six methods.

**Supplementary Tables**

- Supplementary Table S1. The identification performance under different $\alpha$.

- Supplementary Table S2. Significant tests between the MNMO method and the comparison methods (BRCA dataset).

- Supplementary Table S3. Significant tests between the MNMO method and the comparison methods (PRAD dataset).

- Supplementary Table S4. Significant tests between the MNMO method and the comparison methods (LUAD dataset).

- Supplementary Table S5. The terms or pathways that are enriched by the top 30 identified genes.

- Supplementary Table S6. Comparison of identification performance by using different network model (*K*=300).

- Supplementary Table S7. Comparison of identification performance under different combinations of control capacity score, mutation score, and network scores (*K*=300).

- Supplementary Table S8. The number of genes or miRNAs in each layer of the multi-layer heterogeneous network.

- Supplementary Table S9. Comparison of identification performance under different combinations of control capacity score, mutation score, and network scores (*K*=300).

- Supplementary Table S10. Comparison of identification performance under different measurements of network score (*K*=300).

- Supplementary Table S11. The running time of method MNMO on three datasets.

**Supplementary Figures**

**
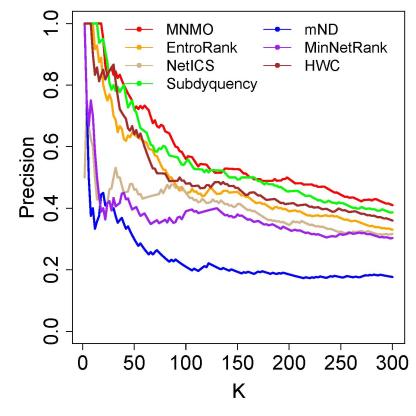

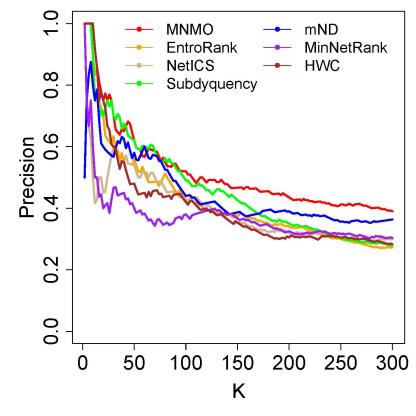

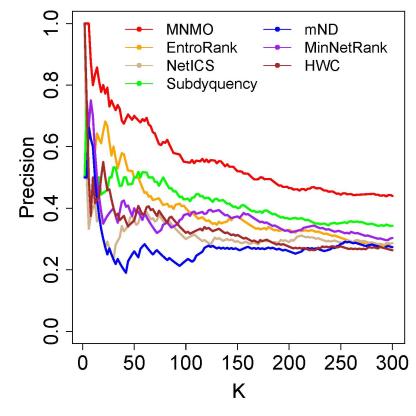
**

(a)BRCA (b)PRAD (c)LUAD

**
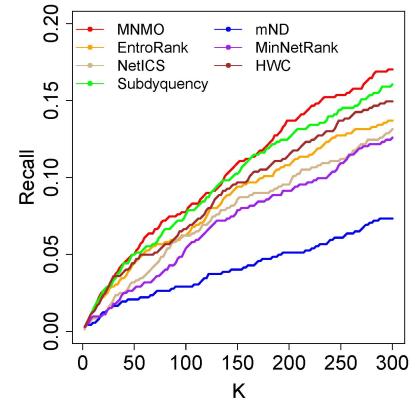

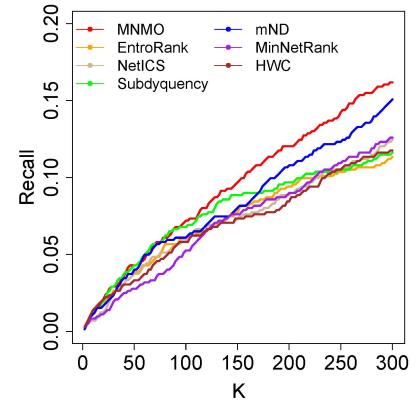

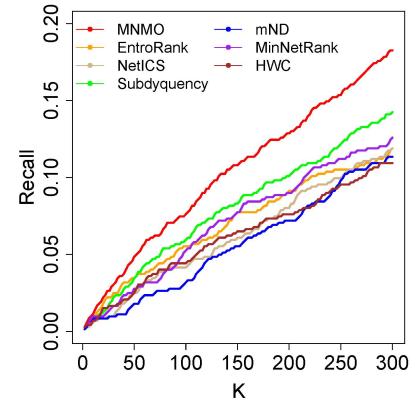
**

(d)BRCA (e)PRAD (f)LUAD

**Supplementary Figure S1. Comparisons of Precision and Recall among MNMO and other six methods.**


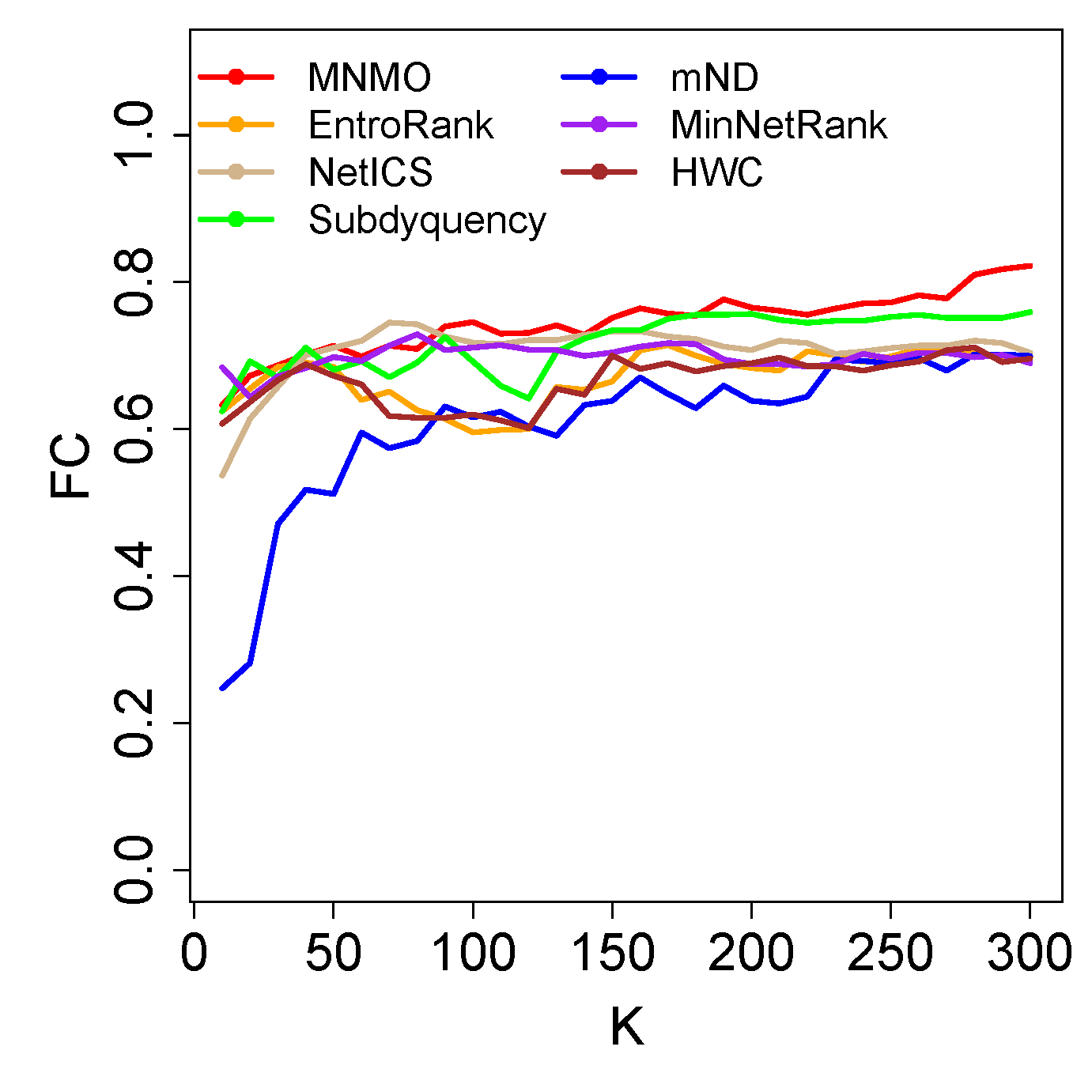

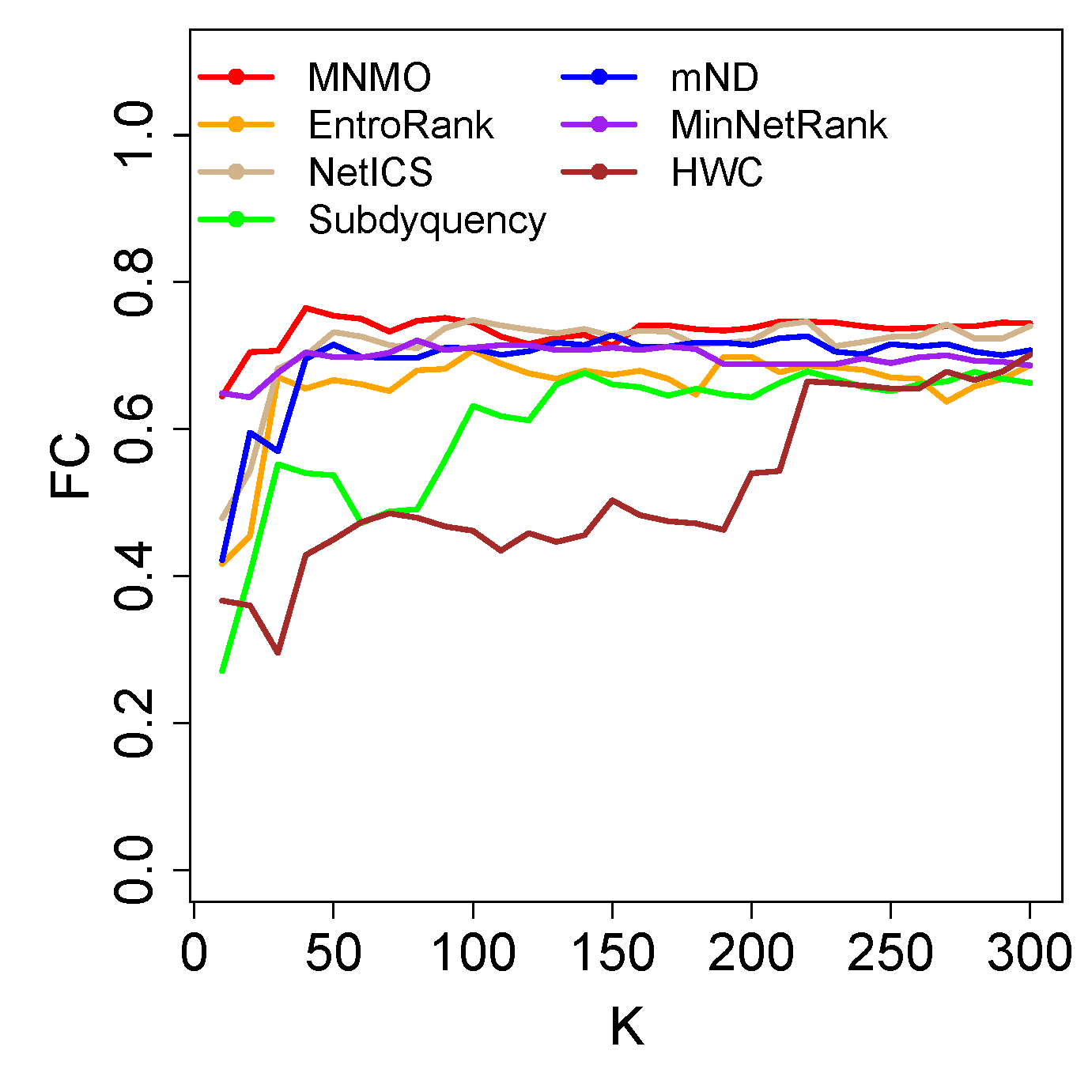

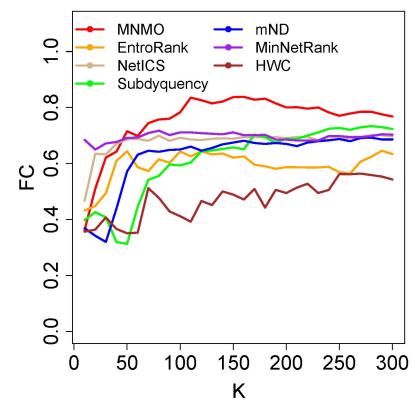


(a)BRCA,KEGG (b)PRAD,KEGG (c)LUAD,KEGG


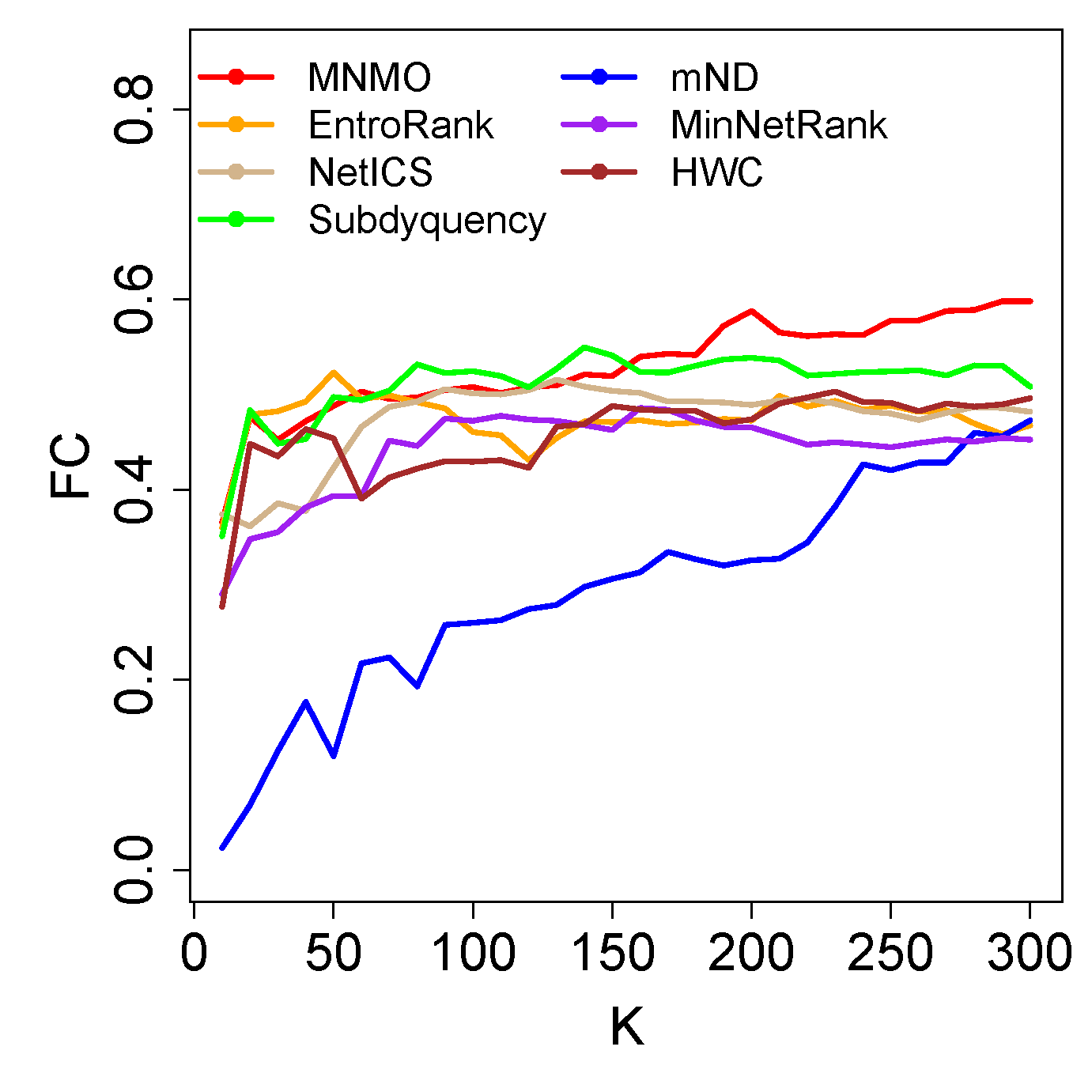

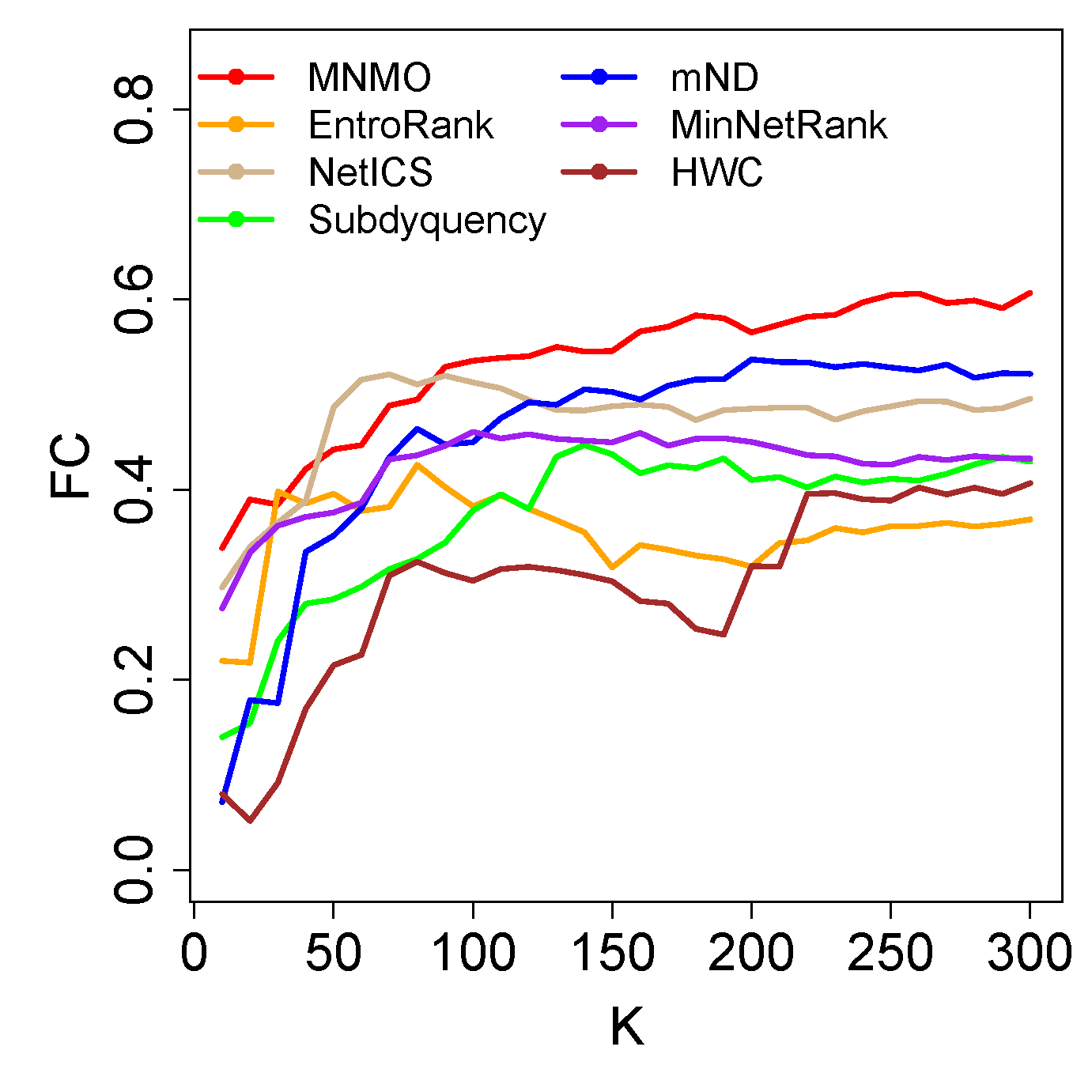

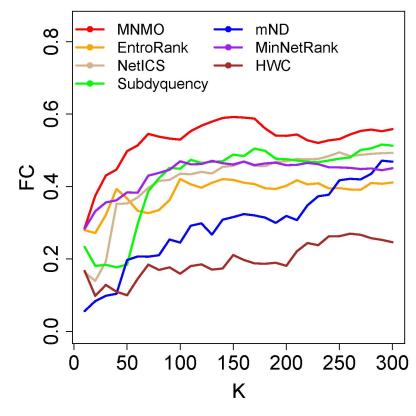


(d)BRCA,REACTOME (e)PRAD,REACTOME (f)LUAD,REACTOME

**Supplementary Figure S2. The comparison results of functional consistency on three cancer datasets.**

**
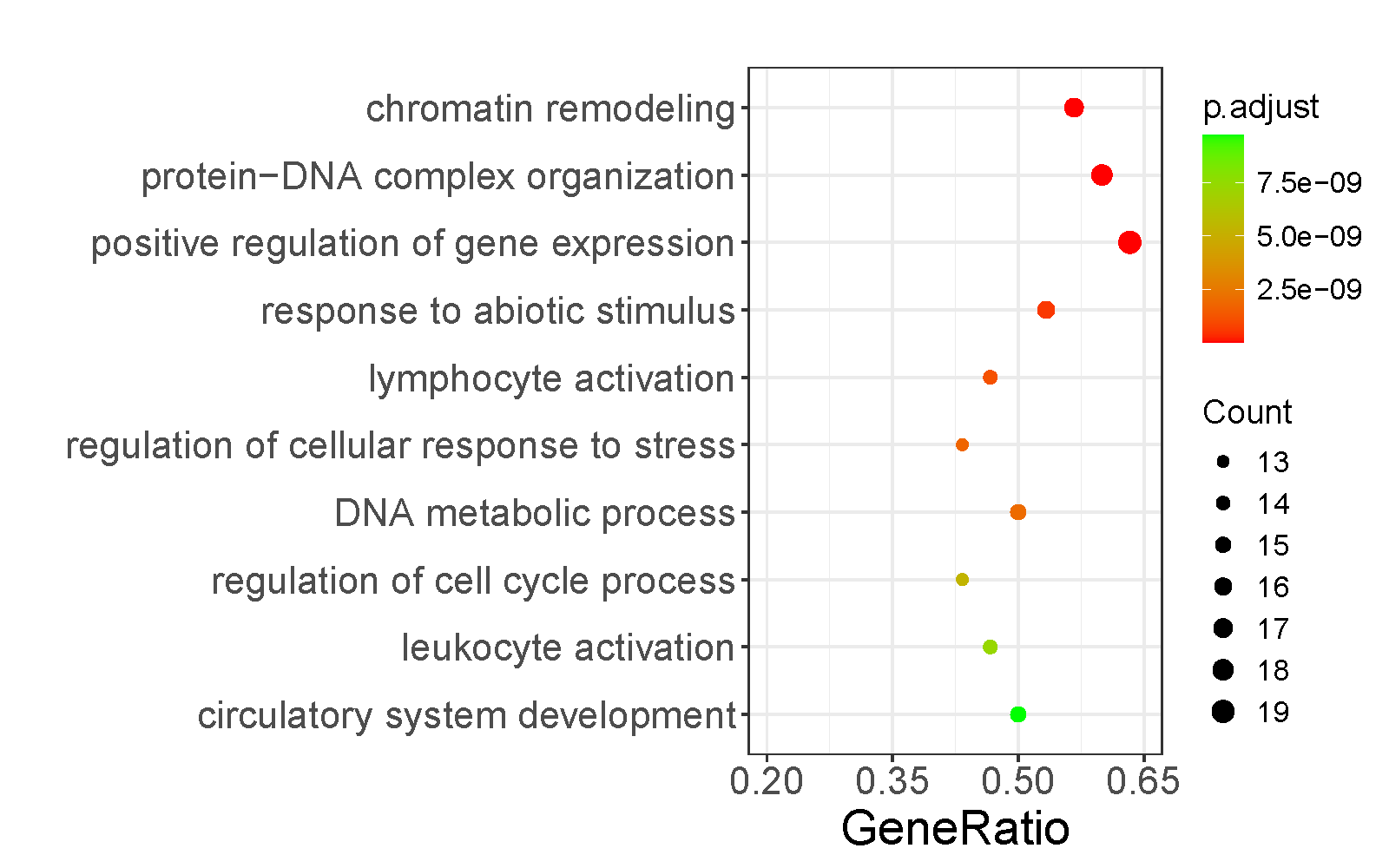

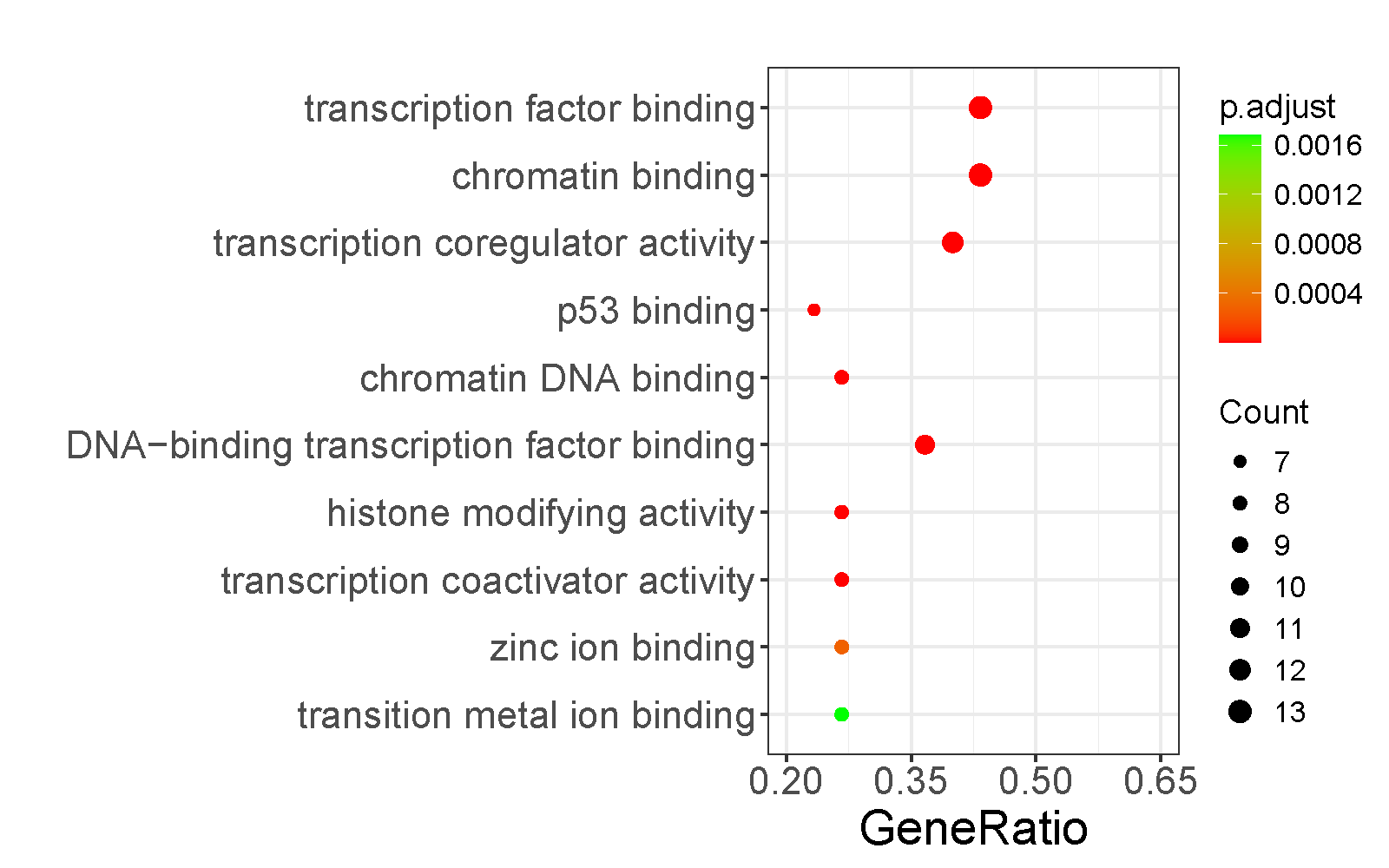
**

1. GO biological process (BP) (b) GO molecular function (MF)

**
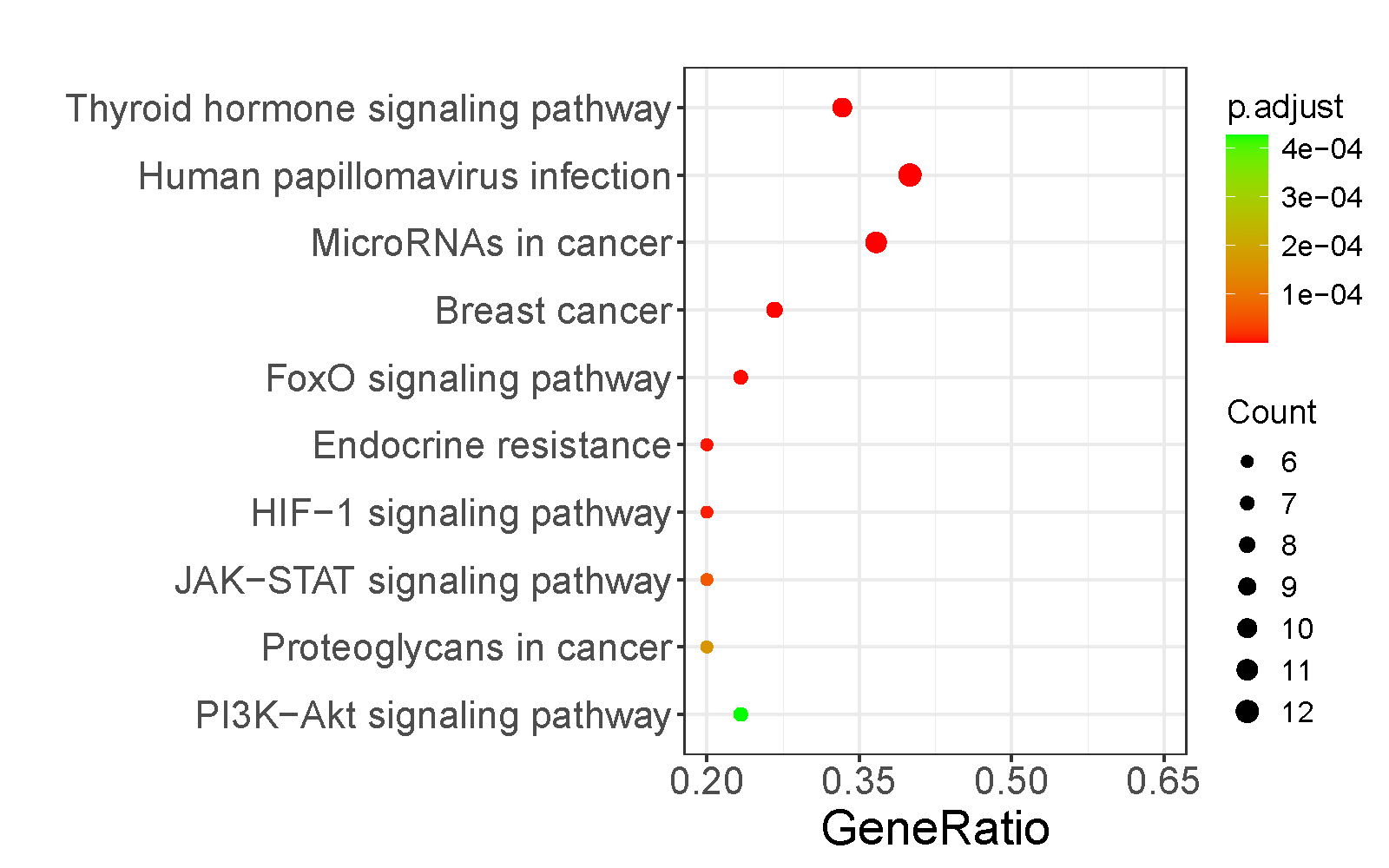

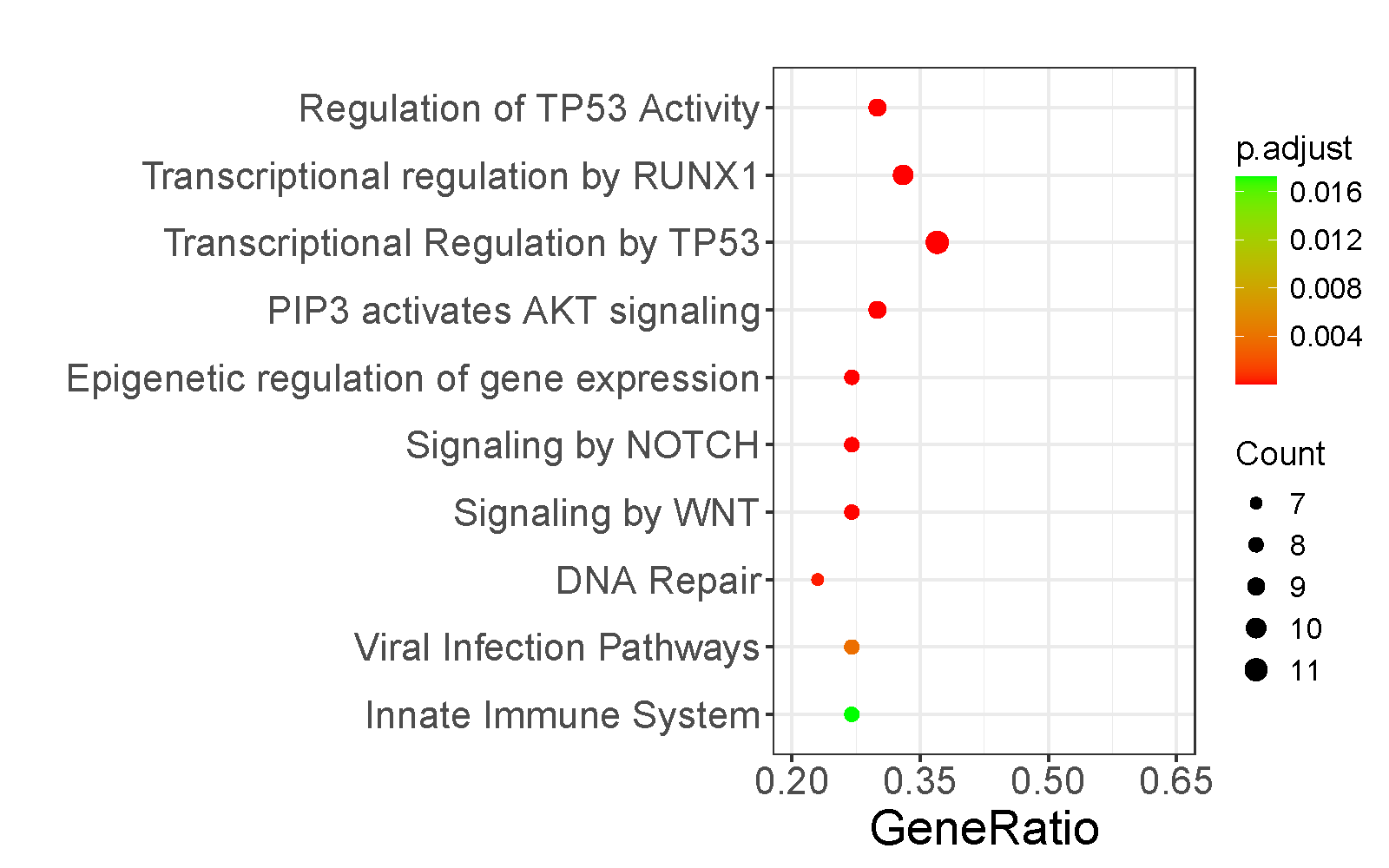
**

(c) KEGG pathway (d) REACTOME pathway

**Supplementary Figure S3. Enrichment analysis on the top 30 genes identified by method MNMO (BRCA dataset).** “Count” indicates the number of genes, “p.adjust” denotes the adjusted *p*-value, which was obtained with R package clusterProfiler. “GeneRadio” measures the ratio of the number of genes associated with a GO term or a pathway to the total number of analyzed genes (i.e., 30). They have the same meaning in the following figures.

**
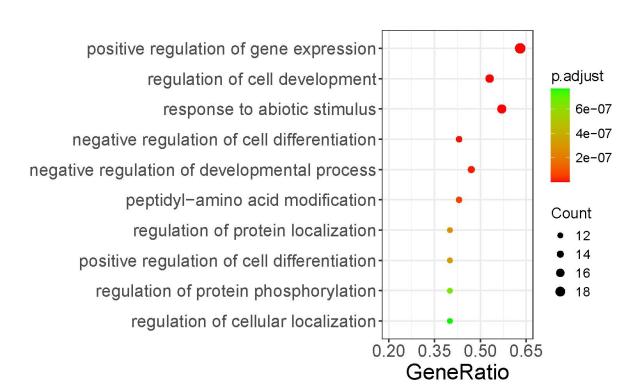

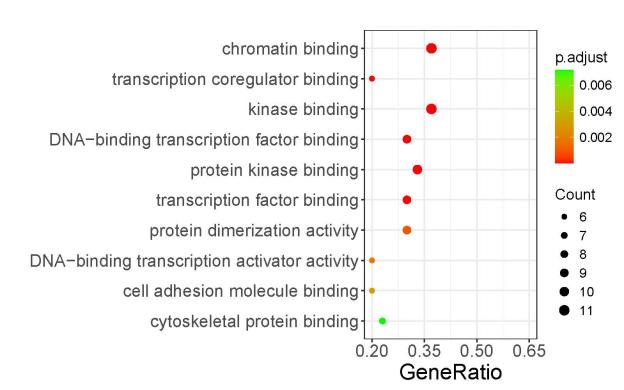
**

(a)GO biological process (BP) (b)GO molecular function (MF)

**
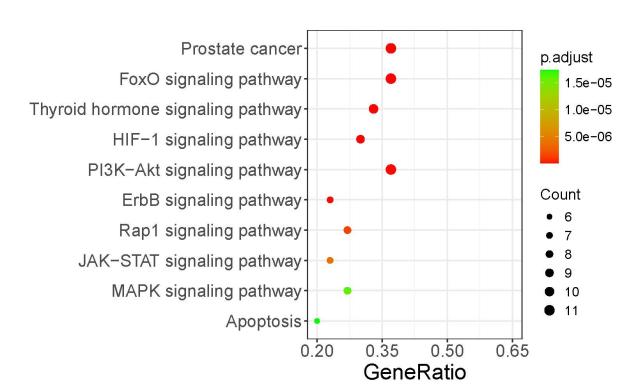

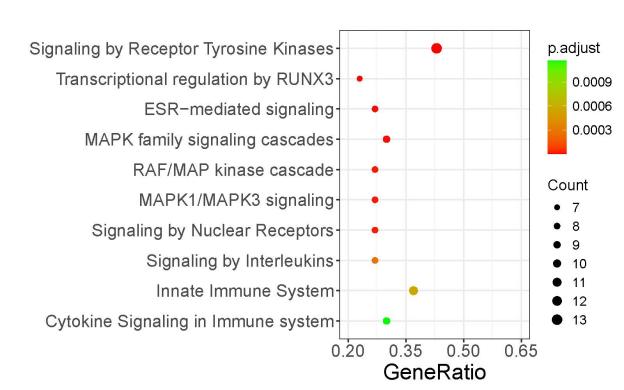
**

(c)KEGG pathway (d)REACTOME pathway

**Supplementary Figure S4. Enrichment analysis on the top 30 genes identified by method MNMO (PRAD dataset).**

**
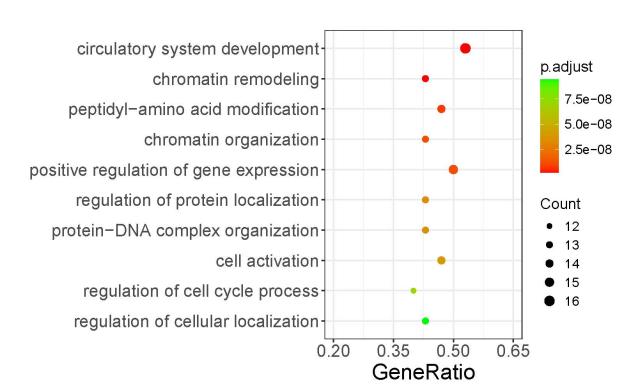

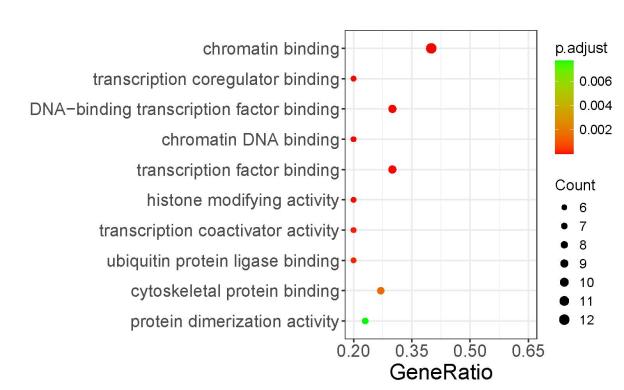
**

(a)GO biological process (BP) (b)GO molecular function (MF)

**
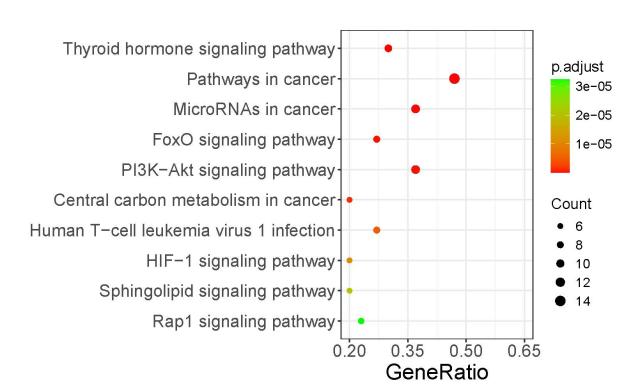

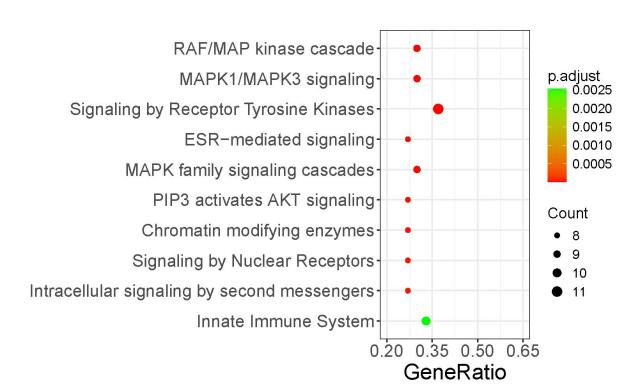
**

(c)KEGG pathway (d)REACTOME pathway

**Supplementary Figure S5. Enrichment analysis on the top 30 genes identified by method MNMO (LUAD dataset).**

**
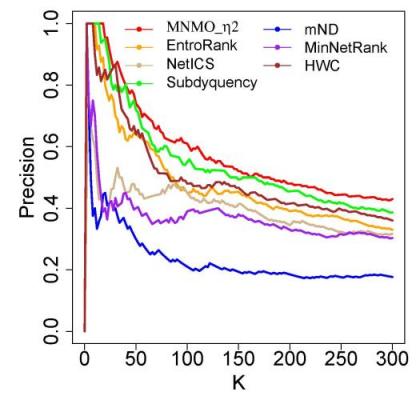

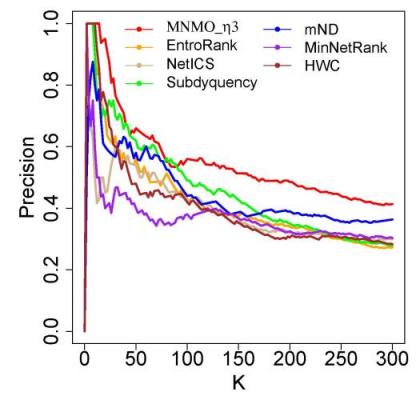

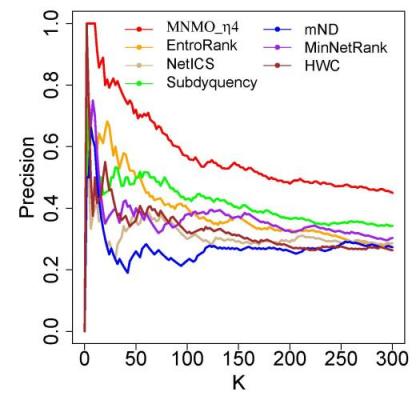
**

(a)BRCA (b)PRAD (c)LUAD

**
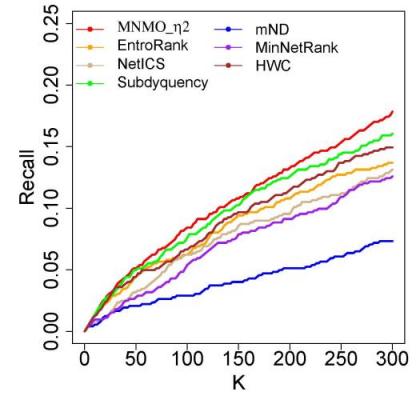

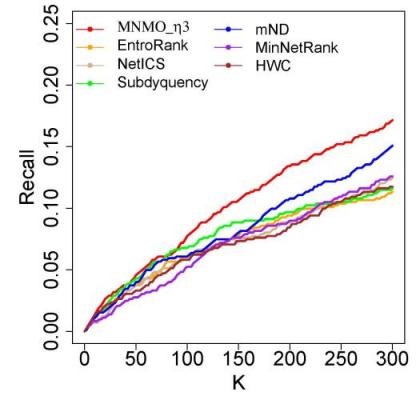

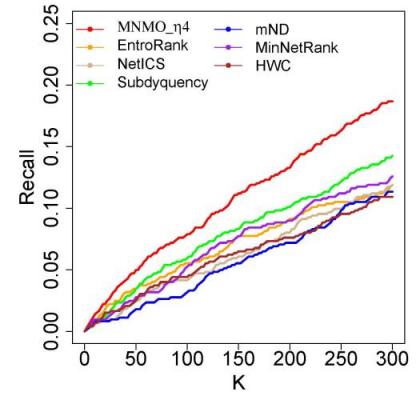
**

(d)BRCA (e)PRAD (f)LUAD

**Supplementary Figure S6. Comparison of Precision and Recall among the extended methods and other six methods.**

**Supplementary Tables**

**Supplementary Table S1. The identification performance under different** $\boldsymbol{\alpha}$**.**

| threshold | $\alpha=$0.1 | $\alpha=$0.5 | $\alpha=$1.0 | $\alpha=$1.5 | $\alpha=2.0$ |
| --- | --- | --- | --- | --- | --- |
|  | BRCA | | | | |
| *Precision* | 0.343 | 0.410 | **0.413** | 0.363 | 0.306 |
| *Recall* | 0.142 | 0.170 | **0.171** | 0.150 | 0.127 |
| *F*1 *score* | 0.201 | 0.240 | **0.242** | 0.213 | 0.179 |
| *FC*(GO) | 0.404 | **0.505** | 0.496 | 0.464 | 0.372 |
| *FC*(KEGG) | 0.769 | **0.811** | 0.784 | 0.784 | 0.707 |
| *FC*(REACTOME) | 0.434 | **0.587** | 0.510 | 0.546 | 0.443 |
| *pAUC* | 0.116 | **0.151** | **0.151** | 0.125 | 0.104 |
|  | PRAD | | | | |
| *Precision* | 0.380 | **0.390** | 0.343 | 0.346 | 0.066 |
| *Recall* | 0.157 | **0.161** | 0.142 | 0.143 | 0.027 |
| *F*1 *score* | 0.222 | **0.228** | 0.201 | 0.203 | 0.039 |
| *FC*(GO) | 0.498 | 0.495 | **0.500** | 0.479 | 0.155 |
| *FC*(KEGG) | 0.754 | 0.743 | **0.770** | 0.708 | 0.046 |
| *FC*(REACTOME) | 0.603 | 0.607 | **0.615** | 0.576 | 0.073 |
| *pAUC* | 0.128 | **0.132** | 0.119 | 0.117 | 0.014 |
|  | LUAD | | | | |
| *Precision* | 0.410 | 0.440 | **0.443** | 0.420 | 0.376 |
| *Recall* | 0.172 | 0.182 | **0.183** | 0.174 | 0.156 |
| *F*1 *score* | 0.244 | 0.258 | **0.260** | 0.246 | 0.220 |
| *FC*(GO) | 0.533 | **0.536** | 0.531 | 0.505 | 0.535 |
| *FC*(KEGG) | 0.758 | 0.766 | **0.815** | 0.774 | 0.692 |
| *FC*(REACTOME) | 0.544 | **0.567** | 0.536 | 0.520 | 0.504 |
| *pAUC* | 0.145 | **0.163** | 0.159 | 0.146 | 0.133 |

**Supplementary Table S2. Significant tests between the MNMO method and the comparison methods (BRCA dataset).**

| Method | *Precision* | *Recall* | *F*1 *score* | *FC*(GO) | *FC*(KEGG) | *FC*(REACTOME) | *pAUC* |
| --- | --- | --- | --- | --- | --- | --- | --- |
| EntroRank | 2.64e-12 | 1.66e-04 | 4.05e-06 | 1.74e-04 | 3.08e-08 | 6.04e-07 | 2.94e-11 |
| NetICS | 8.67e-34 | 2.19e-07 | 2.06e-10 | 0.33 | 4.21e-04 | 1.17e-05 | 1.25e-19 |
| Subdyquency | 0.02 | 0.18 | 0.04 | 0.92 | 0.01 | 0.22 | 0.01 |
| mND | 3.06e-47 | 3.80e-29 | 1.33e-33 | 2.10e-07 | 9.74e-10 | 9.90e-11 | 2.04e-46 |
| MinNetRank | 8.51e-43 | 1.73e-09 | 1.66e-13 | 0.95 | 2.49e-06 | 4.99e-09 | 2.08e-25 |
| HWC | 7.58e-10 | 4.49e-03 | 2.16e-04 | 3.53e-04 | 6.51e-09 | 1.30e-07 | 1.68e-07 |

**Supplementary Table S3. Significant tests between the MNMO method and the comparison methods (PRAD dataset).**

| Method | *Precision* | *Recall* | *F*1 *score* | *FC(*GO*)* | *FC(*KEGG*)* | *FC(*REACTOME*)* | | *pAUC* |
| --- | --- | --- | --- | --- | --- | --- | --- | --- |
| EntroRank | 3.43e-14 | 5.03e-06 | 1.31e-08 | 7.24e-10 | 5.04e-10 | 1.37e-12 | 2.54e-11 | |
| NetICS | 7.04e-17 | 4.44e-06 | 1.17e-08 | 0.02 | 4.73e-03 | 3.02e-05 | 2.16e-12 | |
| Subdyquency | 2.12e-05 | 3.09e-04 | 6.74e-06 | 5.48e-10 | 1.60e-10 | 4.76e-10 | 1.76e-06 | |
| mND | 3.99e-11 | 1.85e-02 | 1.56e-03 | 2.80e-05 | 7.03e-08 | 5.43e-05 | 1.36e-04 | |
| MinNetRank | 2.58e-37 | 2.12e-06 | 5.27e-09 | 1.00e-02 | 5.36e-09 | 2.84e-07 | 3.58e-15 | |
| HWC | 1.71e-18 | 8.49e-07 | 1.22e-09 | 6.00e-13 | 7.34e-11 | 1.98e-13 | 1.72e-13 | |

**Supplementary Table S4. Significant tests between the MNMO method and the comparison methods (LUAD dataset).**

| Method | *Precision* | *Recall* | *F*1 *score* | *FC*(GO) | *FC*(KEGG) | *FC*(REACTOME) | *pAUC* |
| --- | --- | --- | --- | --- | --- | --- | --- |
| EntroRank | 1.43e-28 | 4.37e-10 | 1.20e-13 | 4.22e-07 | 2.19e-08 | 2.16e-11 | 5.34e-21 |
| NetICS | 7.80e-49 | 2.39e-13 | 1.60e-17 | 6.26e-05 | 9.40e-07 | 9.83e-08 | 1.91e-28 |
| Subdyquency | 7.45e-28 | 1.77e-05 | 4.02e-08 | 1.19e-06 | 4.11e-07 | 2.18e-08 | 2.26e-13 |
| mND | 4.33e-46 | 1.72e-16 | 3.29e-21 | 2.74e-08 | 2.78e-07 | 6.00e-13 | 1.57e-33 |
| MinNetRank | 1.30e-43 | 5.05e-09 | 9.24e-13 | 3.53e-04 | 3.31e-06 | 1.73e-08 | 8.92e-22 |
| HWC | 1.23e-44 | 3.54e-14 | 1.54e-18 | 2.04e-12 | 9.57e-10 | 1.69e-17 | 1.21e-28 |

**Supplementary Table S5. The terms or pathways that are enriched by the top 30 identified genes.**

| ID | Term / Pathway | Function description |
| --- | --- | --- |
| GO:0010628 | positive regulation of gene expression | Any process that increases the frequency, rate or extent of gene expression. |
| GO:0006325 | chromatin organization | The assembly or remodeling of chromatin composed of DNA complexed with histones, other associated proteins, and sometimes RNA. |
| GO:0071824 | protein-DNA complex organization | Any process in which macromolecules aggregate, disaggregate, or are modified, resulting in the formation, disassembly, or alteration of a protein-DNA complex. |
| GO:0006338 | Chromatin remodeling | A dynamic process of chromatin reorganization resulting in changes to chromatin structure. These changes allow DNA metabolic processes such as transcriptional regulation, DNA recombination, DNA repair, and DNA replication |
| GO:0009628 | response to abiotic stimulus | Any process that results in a change in state or activity of a cell or an organism (in terms of movement, secretion, enzyme production, gene expression, etc.) as a result of an abiotic (not derived from living organisms) stimulus. |
| GO:0006259 | DNA metabolic process | Any cellular metabolic process involving deoxyribonucleic acid. |
| GO:0072359 | circulatory system development | The process whose specific outcome is the progression of the circulatory system over time, from its formation to the mature structure. |
| GO:0046649 | lymphocyte activation | A change in morphology and behavior of a lymphocyte resulting from exposure to a specific antigen, mitogen, cytokine, chemokine, cellular ligand, or soluble factor. |
| GO:0045321 | leukocyte activation | A change in morphology and behavior of a leukocyte resulting from exposure to a specific antigen, mitogen, cytokine, cellular ligand, or soluble factor. |
| GO:0080135 | regulation of cellular response to stress | Any process that modulates the frequency, rate or extent of a cellular response to stress. |
| GO:0060284 | regulation of cell development | Any process that modulates the rate, frequency or extent of the progression of the cell over time, from its formation to the mature structure. |
| GO:0051093 | negative regulation of developmental process | Any process that stops, prevents or reduces the rate or extent of development. |
| GO:0045596 | negative regulation of cell differentiation | Any process that stops, prevents, or reduces the frequency, rate or extent of cell differentiation. |
| GO:0018193 | peptidyl-amino acid modification | The alteration of an amino acid residue in a peptide. |
| GO:0032880 | regulation of protein localization | Any process that modulates the frequency, rate or extent of any process in which a protein is transported to, or maintained in, a specific location. |
| GO:0045597 | positive regulation of cell differentiation | Any process that activates or increases the frequency, rate or extent of cell differentiation. |
| GO:0001932 | regulation of protein phosphorylation | Any process that modulates the frequency, rate or extent of addition of phosphate groups into an amino acid in a protein. |
| GO:0060341 | regulation of cellular localization | Any process that modulates the frequency, rate or extent of a process in which a cell, a substance, or a cellular entity is transported to, or maintained in a specific location within or in the membrane of a cell. |
| GO:0001775 | cell activation | A multicellular organismal process by which exposure to an activating factor results in a change in the morphology or behavior of a cell. |
| GO:0010564 | regulation of cell cycle process | Any process that modulates a cellular process that is involved in the progression of biochemical and morphological phases and events that occur in a cell during successive cell replication or nuclear replication events. |
| GO:0000278 | mitotic cell cycle | Progression through the phases of the mitotic cell cycle, the most common eukaryotic cell cycle, which canonically comprises four successive phases called G1, S, G2, and M and includes replication of the genome and the subsequent segregation of chromosomes into daughter cells. |
| GO:0035239 | Tube morphogenesis | The process in which the anatomical structures of a tube are generated and organized. |
| GO:0003682 | chromatin binding | Binding to chromatin, the network of fibers of DNA, protein, and sometimes RNA, that make up the chromosomes of the eukaryotic nucleus during interphase. |
| GO:0008134 | transcription factor binding | Binding to a transcription factor, a protein required to initiate or regulate transcription. |
| GO:0003712 | transcription coregulator activity | A transcription regulator activity that modulates the transcription of specific gene sets via binding to a DNA-bound DNA-binding transcription factor. |
| GO:0140297 | DNA-binding transcription factor binding | Binding to a DNA-binding transcription factor, a protein that interacts with a specific DNA sequence within the regulatory region of a gene to modulate transcription. |
| GO:0031490 | chromatin DNA binding | Binding to DNA that is assembled into chromatin. |
| GO:0140993 | histone modifying activity | A catalytic activity that acts on a histone protein. Reversible histone modifications contribute to regulation of gene expression. |
| GO:0003713 | transcription coactivator activity | A transcription coregulator activity that activates or increases the transcription of specific gene sets via binding to a DNA-bound DNA-binding transcription factor. |
| GO:0008270 | zinc ion binding | Binding to a zinc ion (Zn). |
| GO:0046914 | transition metal ion binding | Binding to a transition metal ions. |
| GO:0002039 | p53 binding | Binding to one of the p53 family of proteins. |
| GO:0019900 | kinase binding | Binding to a kinase, any enzyme that catalyzes the transfer of a phosphate group. |
| GO:0019901 | protein kinase binding | Binding to a protein kinase, any enzyme that catalyzes the transfer of a phosphate group, usually from ATP, to a protein substrate. |
| GO:0046983 | protein dimerization activity | The formation of a protein dimer, a macromolecular structure consists of two noncovalently associated identical or nonidentical subunits. |
| GO:0008092 | cytoskeletal protein binding | Binding to a protein component of a cytoskeleton. |
| GO:0001216 | DNA-binding transcription activator activity | A DNA-binding transcription factor activity that activates or increases transcription of specific gene sets. |
| GO:0050839 | cell adhesion molecule binding | Binding to a cell adhesion molecule. |
| GO:0001221 | transcription coregulator binding | Binding to a transcription coregulator. |
| GO:0031625 | ubiquitin protein ligase binding | Binding to a ubiquitin protein ligase enzyme, any of the E3 proteins. |
| hsa05165 | Human papillomavirus infection | Human papillomavirus (HPV) is a non-enveloped, double-stranded DNA virus. HPV infect mucoal and cutaneous epithelium resulting in several types of pathologies. |
| hsa05206 | MicroRNAs in cancer | MicroRNA (miRNA) is a cluster of small non-encoding RNA molecules of 21 - 23 nucleotides in length, which controls gene expression post-transcriptionally either via the degradation of target mRNAs or the inhibition of protein translation. |
| hsa04919 | Thyroid hormone signaling pathway | The thyroid hormones (THs) are important regulators of growth, development and metabolism. |
| hsa05224 | Breast cancer | Breast cancer is the leading cause of cancer death among women worldwide. The vast majority of breast cancers are carcinomas that originate from cells lining the milk-forming ducts of the mammary gland. |
| hsa04068 | FoxO signaling pathway | The forkhead box O (FOXO) family of transcription factors regulates the expression of genes in cellular physiological events including apoptosis, cell-cycle control, glucose metabolism, oxidative stress resistance, and longevity. |
| hsa04151 | PI3K-Akt signaling pathway | The phosphatidylinositol 3' -kinase(PI3K)-Akt signaling pathway is activated by many types of cellular stimuli or toxic insults and regulates fundamental cellular functions such as transcription, translation, proliferation, growth, and survival. |
| hsa01522 | Endocrine resistance | Endocrine therapy is a key treatment strategy to control or eradicate hormone-responsive breast cancer. Mechanisms of endocrine resistance include loss of ER-alpha expression, altered expression of coactivators or coregulators that play a critical role in ER-mediated gene transcription. |
| hsa04066 | HIF-1 signaling pathway | Hypoxia-inducible factor 1 (HIF-1) is a transcription factor that functions as a master regulator of oxygen homeostasis. |
| hsa04630 | JAK-STAT signaling pathway | The Janus kinase/signal transducers and activators of transcription (JAK/STAT) pathway is one of a handful of pleiotropic cascades used to transduce a multitude of signals for development and homeostasis in animals. |
| hsa05205 | Proteoglycans in cancer | Many proteoglycans (PGs) in the tumor microenvironment have been shown to be key macromolecules that contribute to biology of various types of cancer including proliferation, adhesion, angiogenesis and metastasis, affecting tumor progress. |
| hsa05215 | Prostate cancer | Prostate cancer constitutes a major health problem in Western countries. It is the most frequently diagnosed cancer among men and the second leading cause of male cancer deaths. |
| hsa04015 | Rap1 signaling pathway | Rap1 is a small GTPase that controls diverse processes, such as cell adhesion, cell-cell junction formation and cell polarity. |
| hsa04010 | MAPK signaling pathway | The mitogen-activated protein kinase (MAPK) cascade is a highly conserved module that is involved in various cellular functions, including cell proliferation, differentiation and migration. |
| hsa04012 | ErbB signaling pathway | The ErbB family of receptor tyrosine kinases (RTKs) couples binding of extracellular growth factor ligands to intracellular signaling pathways regulating diverse biologic responses, including proliferation, differentiation, cell motility, and survival. |
| hsa04210 | Apoptosis | Apoptosis is a genetically programmed process for the elimination of damaged or redundant cells by activation of caspases (aspartate-specific cysteine proteases). |
| hsa05166 | Human T-cell leukemia virus 1 infection | Human T-cell leukemia virus type 1 (HTLV-1) is a pathogenic retrovirus that is associated with adult T-cell leukemia/lymphoma (ATL). |
| hsa05230 | Central carbon metabolism in cancer | Malignant transformation of cells requires specific adaptations of cellular metabolism to support growth and survival. |
| hsa04071 | Sphingolipid signaling pathway | Sphingomyelin (SM) and its metabolic products are now known to have second messenger functions in a variety of cellular signaling pathways. |
| R-HSA-4839726 | Chromatin organization | Chromatin organization refers to the composition and conformation of complexes between DNA, protein and RNA. |
| R-HSA-3700989 | Transcriptional Regulation by TP53 | The tumor suppressor TP53 is a transcription factor. Under stress conditions, it recognizes specific responsive DNA elements and thus regulates the transcription of many genes involved in a variety of cellular processes. |
| R-HSA-8878171 | Transcriptional regulation by RUNX1 | The RUNX1 transcription factor is a master regulator of hematopoiesis. RUNX1 loss-of-function decreases ribosome biogenesis and translation in hematopoietic stem and progenitor cells (HSPCs). |
| R-HSA-5633007 | Regulation of TP53 Activity | Protein stability and transcriptional activity of TP53 (p53) tumor suppressor are regulated by post-translational modifications. |
| R-HSA-1257604 | PIP3 activates AKT signaling | Signaling by AKT is one of the key outcomes of receptor tyrosine kinase (RTK) activation. AKT is activated by the cellular second messenger , a phospholipid that is generated by PI3K. |
| R-HSA-212165 | Epigenetic regulation of gene expression | Epigenetic processes regulate gene expression by modulating the frequency, rate. |
| R-HSA-157118 | Signaling by NOTCH | The Notch Signaling Pathway (NSP) is a highly conserved pathway for cell-cell communication. NSP is involved in the regulation of cellular differentiation, proliferation, and specification. |
| R-HSA-195721 | Signaling by WNT | WNT signaling pathways control a wide range of developmental and adult process in metozoans including cell proliferation, cell fate decisions, cell polarity and stem cell maintenance |
| R-HSA-73894 | DNA Repair | DNA repair is a phenomenal multi-enzyme, multi-pathway system required to ensure the integrity of the cellular genome. |
| R-HSA-168249 | Innate Immune System | Innate immunity encompases the nonspecific part of immunity tha are part of an individual's natural biologic makeup. |
| R-HSA-5683057 | MAPK family signaling cascades | The mitogen activated protein kinases (MAPKs) are a family of conserved protein serine threonine kinases that respond to varied extracellular stimuli to activate intracellular processes including gene expression, metabolism and apoptosis, among others. |
| R-HSA-1280215 | Cytokine Signaling in Immune system | Cytokines are small proteins that regulate and mediate immunity, inflammation, and hematopoiesis. |
| R-HSA-8939211 | ESR-mediated signaling | Estrogens are a class of hormones that play a role in physiological processes such as development, reproduction, metabolism of liver, fat and bone, and neuronal and cardiovascular function. |
| R-HSA-5673001 | RAF/MAP kinase cascade | The RAS-RAF-MEK-ERK pathway regulates processes such as proliferation, differentiation, survival, senescence and cell motility in response to growth factors, hormones and cytokines, among others. |
| R-HSA-5684996 | MAPK1/MAPK3 signaling | MAPK3 and MAPK1 are phosphorylated by the MAP2Ks 1 and 2 in response to a wide range of extracellular stimuli to promote differentiation, proliferation, cell motility, cell survivial, metabolism and transcription, among others. |
| R-HSA-9006934 | Signaling by Receptor Tyrosine Kinases | Receptor tyrosine kinases (RTKs) are a major class of cell surface proteins involved in Signal Transduction. |
| R-HSA-9006931 | Signaling by Nuclear Receptors | Nuclear receptors (NRs) are ligand-activated transcription factors that bind to small lipid based molecules to regulate gene expression and other cellular process. |
| R-HSA-449147 | Signaling by Interleukins | Interleukins are low molecular weight proteins that bind to cell surface receptors and act in an autocrine and/or paracrine fashion. |
| R-HSA-8878159 | Transcriptional regulation by RUNX3 | The transcription factor RUNX3 is a RUNX family member. |
| R-HSA-9006925 | Intracellular signaling by second messengers | Second messengers are generated within the cell as a downstream step in signal transduction cascades initiated by the interaction of an external stimulus with a cell surface receptor. |

(The function descriptions are extracted from databases GO, KEGG, and REACTOME, respectively)

**Supplementary Table S6. Comparison of identification performance by using different network model (*K*=300).**

|  | $\mathbf{MNMO}$ | $\boldsymbol{MNMO\_1}$ | $\boldsymbol{MNMO\_}$**2** | $\boldsymbol{MNMO\_}$**3** | $\boldsymbol{MNMO\_}$**4** |
| --- | --- | --- | --- | --- | --- |
|  | BRCA | | | | |
| *Precision* | **0.410** | 0.376 | 0.390 | 0.380 | 0.403 |
| *Recall* | **0.170** | 0.156 | 0.161 | 0.157 | 0.167 |
| *F*1 *score* | **0.240** | 0.220 | 0.228 | 0.222 | 0.236 |
| *FC*(GO) | **0.505** | 0.493 | 0.498 | 0.495 | 0.499 |
| *FC*(KEGG) | **0.811** | 0.785 | 0.770 | 0.781 | 0.784 |
| *FC*(REACTOME) | **0.587** | 0.583 | 0.566 | 0.585 | 0.586 |
| *pAUC* | **0.151** | 0.128 | 0.140 | 0.130 | 0.144 |
|  | PRAD | | | | |
| *Precision* | **0.390** | 0.346 | 0.346 | 0.356 | 0.356 |
| *Recall* | **0.161** | 0.143 | 0.143 | 0.147 | 0.147 |
| *F*1 *score* | **0.228** | 0.203 | 0.203 | 0.209 | 0.209 |
| *FC*(GO) | **0.495** | 0.458 | 0.464 | 0.464 | 0.468 |
| *FC*(KEGG) | **0.743** | 0.691 | 0.688 | 0.710 | 0.698 |
| *FC*(REACTOME) | **0.607** | 0.561 | 0.571 | 0.567 | 0.568 |
| *pAUC* | **0.132** | 0.112 | 0.115 | 0.121 | 0.122 |
|  | LUAD | | | | |
| *Precision* | **0.440** | 0.350 | 0.363 | 0.353 | 0.386 |
| *Recall* | **0.182** | 0.145 | 0.150 | 0.146 | 0.160 |
| *F*1 *score* | **0.258** | 0.205 | 0.213 | 0.207 | 0.226 |
| *FC*(GO) | **0.536** | 0.489 | 0.486 | 0.486 | 0.486 |
| *FC*(KEGG) | **0.766** | 0.704 | 0.706 | 0.712 | 0.716 |
| *FC*(REACTOME) | 0.567 | 0.529 | 0.546 | **0.582** | 0.546 |
| *pAUC* | **0.163** | 0.115 | 0.129 | 0.122 | 0.137 |

**Supplementary Table S7.** **Comparison of identification performance under different combinations of control capacity score, mutation score, and network scores (*K*=300).**

|  | $\mathbf{MNMO}$ | $\boldsymbol{MNMO\_}$**C** | $\boldsymbol{MNMO\_}$**M** | $\boldsymbol{MNMO\_}$**N** | $\boldsymbol{MNMO\_}$**MN** | $\boldsymbol{MNMO\_}$**CN** | $\boldsymbol{MNMO\_}$**CM** |
| --- | --- | --- | --- | --- | --- | --- | --- |
|  | BRCA | | | | | | |
| *Precision* | **0.410** | 0.220 | 0.283 | 0.236 | 0.403 | 0.333 | 0.376 |
| *Recall* | **0.170** | 0.091 | 0.117 | 0.098 | 0.167 | 0.138 | 0.156 |
| *F*1 *score* | **0.240** | 0.129 | 0.166 | 0.138 | 0.236 | 0.195 | 0.220 |
| *FC*(GO) | **0.505** | 0.186 | 0.187 | 0.425 | 0.481 | 0.497 | 0.333 |
| *FC*(KEGG) | **0.811** | 0.294 | 0.325 | 0.707 | 0.774 | 0.737 | 0.484 |
| *FC*(REACTOME) | **0.587** | 0.294 | 0.220 | 0.428 | 0.558 | 0.467 | 0.378 |
| *pAUC* | **0.151** | 0.063 | 0.092 | 0.069 | 0.145 | 0.106 | 0.129 |
|  | PRAD | | | | | | |
| *Precision* | 0.390 | 0.126 | 0.260 | 0.300 | **0.406** | 0.276 | 0.326 |
| *Recall* | 0.16**1** | 0.052 | 0.107 | 0.124 | **0.168** | 0.114 | 0.135 |
| *F*1 *score* | 0.228 | 0.074 | 0.152 | 0.175 | **0.238** | 0.162 | 0.191 |
| *FC*(GO) | 0.495 | 0.316 | 0.221 | 0.474 | **0.499** | 0.459 | 0.450 |
| *FC*(KEGG) | 0.743 | 0.552 | 0.367 | 0.725 | **0.767** | 0.675 | 0.715 |
| *FC*(REACTOME) | **0.607** | 0.232 | 0.099 | 0.478 | 0.597 | 0.507 | 0.502 |
| *pAUC* | 0.132 | 0.035 | 0.074 | 0.095 | **0.143** | 0.090 | 0.104 |
|  | LUAD | | | | | | |
| *Precision* | **0.440** | 0.230 | 0.143 | 0.263 | 0.410 | 0.343 | 0.346 |
| *Recall* | **0.182** | 0.095 | 0.059 | 0.109 | 0.170 | 0.142 | 0.143 |
| *F*1 *score* | **0.258** | 0.134 | 0.084 | 0.154 | 0.240 | 0.201 | 0.203 |
| *FC*(GO) | **0.536** | 0.196 | 0.060 | 0.448 | 0.482 | 0.492 | 0.383 |
| *FC*(KEGG) | **0.766** | 0.202 | 0.186 | 0.688 | 0.704 | 0.765 | 0.596 |
| *FC*(REACTOME) | 0.567 | 0.292 | 0.026 | 0.458 | **0.595** | 0.470 | 0.387 |
| *pAUC* | **0.163** | 0.062 | 0.034 | 0.081 | 0.141 | 0.108 | 0.114 |

**Supplementary Table S8. The number of genes or miRNAs in each layer of the multi-layer heterogeneous network.**

| Dataset | miRNAs (layer one) | genes (layer two) | genes (layer three) | genes (layer four) |
| --- | --- | --- | --- | --- |
| BRCA | 190 | 12517 | 1862 | 458 |
| LUAD | 388 | 11675 | 1224 | 438 |
| LIHC | 282 | 9998 | 3309 | 853 |
| LUSC | 533 | 12538 | 1108 | 389 |
| KIRP | 152 | 8329 | 1101 | 904 |
| GBM | 567 | 8410 | 6152 | 1416 |
| PRAD | 69 | 8020 | 427 | 386 |
| COAD | 485 | 9703 | 398 | 55 |

**Supplementary Table S9. Comparison of identification performance under different combinations of control capacity score, mutation score, and network scores (*K*=300).**

|  | $\mathbf{MNMO}$ | $\boldsymbol{MNMO\_}$**C** | $\boldsymbol{MNMO\_}$**M** | $\boldsymbol{MNMO\_}$**N** | $\boldsymbol{MNMO\_}$**MN** | $\boldsymbol{MNMO\_}$**CN** | $\boldsymbol{MNMO\_}$**CM** |
| --- | --- | --- | --- | --- | --- | --- | --- |
|  | LIHC | | | | | | |
| *Precision* | **0.406** | 0.220 | 0.260 | 0.290 | 0.363 | 0.313 | 0.363 |
| *Recall* | **0.168** | 0.091 | 0.107 | 0.120 | 0.150 | 0.130 | 0.150 |
| *F*1 *score* | **0.238** | 0.129 | 0.152 | 0.170 | 0.213 | 0.183 | 0.213 |
| *FC*(GO) | **0.506** | 0.374 | 0.234 | 0.444 | 0.486 | 0.484 | 0.441 |
| *FC*(KEGG) | **0.798** | 0.733 | 0.435 | 0.721 | 0.759 | 0.741 | 0.757 |
| *FC*(REACTOME) | 0.580 | 0.437 | 0.333 | 0.451 | **0.594** | 0.487 | 0.571 |
| *pAUC* | **0.143** | 0.065 | 0.076 | 0.089 | 0.129 | 0.102 | 0.118 |
|  | LUSC | | | | | | |
| *Precision* | **0.393** | 0.21 | 0.173 | 0.243 | 0.343 | 0.290 | 0.266 |
| *Recall* | **0.163** | 0.087 | 0.071 | 0.100 | 0.142 | 0.120 | 0.110 |
| *F*1 *score* | **0.224** | 0.074 | 0.142 | 0.150 | 0.199 | 0.191 | 0.183 |
| *FC*(GO) | **0.511** | 0.218 | 0.074 | 0.449 | 0.468 | 0.476 | 0.297 |
| *FC*(KEGG) | 0.745 | 0.521 | 0.102 | 0.691 | 0.710 | **0.753** | 0.520 |
| *FC*(REACTOME) | 0.537 | 0.341 | 0.028 | 0.450 | **0.595** | 0.452 | 0.321 |
| *pAUC* | **0.136** | 0.057 | 0.043 | 0.073 | 0.114 | 0.090 | 0.084 |
|  | KIRP | | | | | | |
| *Precision* | **0.383** | 0.126 | 0.243 | 0.256 | 0.340 | 0.326 | 0.313 |
| *Recall* | **0.159** | 0.052 | 0.100 | 0.106 | 0.141 | 0.135 | 0.130 |
| *F*1 *score* | **0.258** | 0.134 | 0.084 | 0.154 | 0.240 | 0.201 | 0.203 |
| *FC*(GO) | **0.493** | 0.267 | 0.200 | 0.436 | 0.442 | 0.487 | 0.404 |
| *FC*(KEGG) | 0.767 | 0.554 | 0.275 | 0.719 | **0.777** | 0.710 | 0.674 |
| *FC*(REACTOME) | **0.522** | 0.265 | 0.258 | 0.445 | 0.487 | 0.468 | 0.409 |
| *pAUC* | **0.132** | 0.036 | 0.076 | 0.079 | 0.119 | 0.103 | 0.107 |
|  | GBM | | | | | | |
| *Precision* | **0.350** | 0.200 | 0.230 | 0.260 | 0.340 | 0.290 | 0.293 |
| *Recall* | **0.145** | 0.082 | 0.095 | 0.107 | 0.141 | 0.120 | 0.121 |
| *F*1 *score* | **0.205** | 0.117 | 0.134 | 0.152 | 0.199 | 0.170 | 0.172 |
| *FC*(GO) | **0.469** | 0.248 | 0.174 | 0.427 | 0.465 | 0.445 | 0.358 |
| *FC*(KEGG) | **0.735** | 0.639 | 0.385 | 0.679 | 0.728 | 0.695 | 0.704 |
| *FC*(REACTOME) | 0.517 | 0.314 | 0.261 | 0.432 | 0.520 | 0.457 | **0.537** |
| *pAUC* | **0.117** | 0.053 | 0.064 | 0.080 | 0.112 | 0.090 | 0.101 |
|  | COAD | | | | | | |
| *Precision* | 0.363 | 0.203 | 0.243 | 0.230 | **0.370** | 0.316 | 0.313 |
| *Recall* | 0.150 | 0.084 | 0.100 | 0.095 | **0.153** | 0.131 | 0.130 |
| *F*1 *score* | 0.213 | 0.119 | 0.142 | 0.134 | **0.217** | 0.185 | 0.183 |
| *FC*(GO) | 0.480 | 0.176 | 0.228 | 0.459 | **0.502** | 0.461 | 0.354 |
| *FC*(KEGG) | **0.786** | 0.485 | 0.355 | 0.693 | 0.726 | 0.772 | 0.672 |
| *FC*(REACTOME) | **0.567** | 0.255 | 0.233 | 0.448 | 0.534 | 0.513 | 0.425 |
| *pAUC* | 0.127 | 0.059 | 0.071 | 0.068 | **0.131** | 0.102 | 0.100 |

**Supplementary Table S10.** **Comparison of identification performance under different measurements of network score (*K*=300).**

|  | $\mathbf{MNMO}$ | $\boldsymbol{MNMO\_}\boldsymbol{\eta}\mathbf{1}$ | $\boldsymbol{MNMO\_}\boldsymbol{\eta}$**2** | $\boldsymbol{MNMO\_}\boldsymbol{\eta}$**3** | $\boldsymbol{MNMO\_}\boldsymbol{\eta}$**4** |
| --- | --- | --- | --- | --- | --- |
|  | BRCA | | | | |
| *Precision* | 0.410 | 0.400 | **0.430** | 0.403 | 0.406 |
| *Recall* | 0.170 | 0.165 | **0.178** | 0.167 | 0.168 |
| *F*1 *score* | 0.240 | 0.234 | **0.252** | 0.236 | 0.238 |
| *FC*(GO) | **0.505** | 0.456 | 0.500 | 0.441 | 0.468 |
| *FC*(KEGG) | **0.811** | 0.747 | 0.787 | 0.750 | 0.774 |
| *FC*(REACTOME) | **0.587** | 0.540 | 0.580 | 0.516 | 0.573 |
| *pAUC* | **0.151** | 0.141 | 0.150 | 0.141 | 0.148 |
|  | PRAD | | | | |
| *Precision* | 0.390 | 0.410 | 0.373 | **0.413** | 0.396 |
| *Recall* | 0.161 | 0.170 | 0.154 | **0.171** | 0.164 |
| *F*1 *score* | 0.228 | 0.240 | 0.218 | **0.242** | 0.232 |
| *FC*(GO) | 0.495 | 0.519 | 0.497 | **0.522** | 0.502 |
| *FC*(KEGG) | 0.743 | 0.757 | 0.733 | **0.778** | 0.743 |
| *FC*(REACTOME) | 0.607 | 0.624 | 0.604 | **0.631** | 0.613 |
| *pAUC* | 0.132 | **0.144** | 0.130 | 0.143 | 0.138 |
|  | LUAD | | | | |
| *Precision* | 0.440 | 0.443 | 0.443 | 0.440 | **0.450** |
| *Recall* | 0.182 | 0.183 | 0.183 | 0.182 | **0.186** |
| *F*1 *score* | 0.258 | 0.260 | 0.260 | 0.258 | **0.263** |
| *FC*(GO) | 0.536 | 0.532 | 0.530 | 0.531 | **0.542** |
| *FC*(KEGG) | 0.766 | 0.803 | 0.816 | **0.831** | 0.795 |
| *FC*(REACTOME) | **0.567** | 0.559 | 0.562 | 0.553 | 0.559 |
| *pAUC* | **0.163** | 0.162 | 0.162 | **0.163** | **0.163** |

**Supplementary Table S11. The running time of method MNMO on three datasets .**

| Datasets | Time (mins) |
| --- | --- |
| Data1 | 76.5 |
| Data2 | 25.1 |
| Data3 | 50.3 |
